# From green chemistry to biomedicine: the sustainable symphony of cobalt oxide nanoparticles

**DOI:** 10.1039/d4ra05872k

**Published:** 2024-10-17

**Authors:** Annu, Muskan Sahu, Somesh Singh, Satypal Prajapati, Dinesh K. Verma, Dong Kil Shin

**Affiliations:** a Materials Laboratory, School of Mechanical Engineering, Yeungnam University 280 Daehak-ro Gyeongsan-si Gyeongsanbuk-do 38541 Republic of Korea annuchem92@gmail.com dkshin@yu.ac.kr; b Department of Chemistry, Prof. Rajendra Singh (Rajju Bhaiya) Institute of Physical Sciences for Study and Research, V.B.S. Purvanchal University Jaunpur-222003 India dineshkv.rs.chy15@iitbhu.ac.in

## Abstract

Deciphering the importance of nanostructures in advanced technologies for a broad application spectrum has far-reaching implications for humans and the environment. Cost-effective, abundant cobalt oxide nanoparticles (NPs) are among the most attractive and extensively utilized materials in biomedical sciences due to their high chemical stability, and biocompatibility. However, the methods used to develop the NPs are hazardous for human health and the environment. This article precisely examines diverse green synthesis methods employing plant extracts and microbial sources, shedding light on their mechanism, and eco-friendly attributes with more emphasis on biocompatible properties accompanied by their challenges and avenues for further research. An in-depth analysis of the synthesized cobalt oxide NPs by various characterization techniques reveals their multifaceted functionalities including cytotoxicity, larvicidal, antileishmanial, hemolytic, anticoagulating, thrombolytic, anticancer and drug sensing abilities. This revelatory and visionary article helps researchers to contribute to advancing sustainable practices in nanomaterial synthesis and illustrates the potential of biogenically derived cobalt oxide NPs in fostering green and efficient technologies for biomedical applications.

## Abstract 2

Unlocking the potential of green-synthesized cobalt oxide nanoparticles: bridging sustainable chemistry with cutting-edge biomedical innovations.

## Graphical abstract

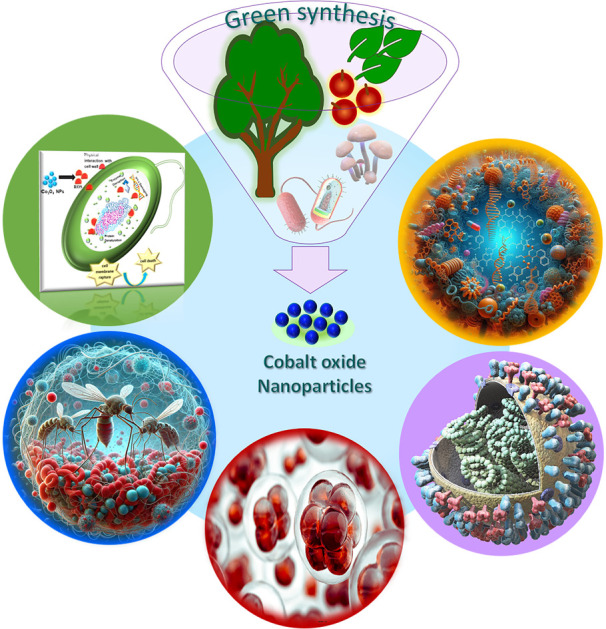

## 1. Introduction

With the advancement of interdisciplinary research in nanoscience, there is a growing need for producing NPs through sustainable and eco-friendly methods to prevent harm to the environment and nearby mammalian communities. Nanotechnologists are thus developing controlled, eco-friendly approaches for NP synthesis, leveraging their small size, porosity, and large surface-to-volume ratio for applications in medicine, catalysis, industry, and environmental remediation.^1–6^ Traditionally, harmful chemicals were used, raising environmental concerns. However, with the NP market projected to grow by 2026, green synthesis using plant-derived phytochemicals is becoming pivotal, aligning with United Nations sustainable development goals.^7,8^

Metal oxide NPs are widely used in industries such as building materials, pharmaceuticals, cosmetics, textiles, electronics, environmental protection, and renewable energy.^8–14^ Common metal oxides like ZnO, Al_2_O_3_, TiO_2_, NiO, CeO_2_, CuO, and MgO exhibit significant toxicity, causing cytotoxicity, oxidative stress, DNA damage, and inflammation. For instance, ZnO NPs are particularly harmful to human pulmonary cells, and TiO_2_ NPs cause DNA damage.^15,16^ In contrast, cobalt oxide (Co_3_O_4_) NPs are less toxic and environmentally benign, making them attractive for various scientific and technological applications due to their cost-effectiveness and eco-friendly properties.^17^

**Fig. 1:**
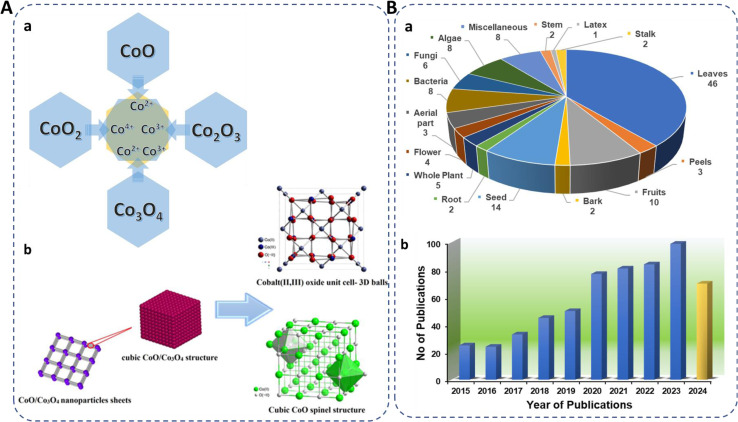
(A) (a) Existing forms of cobalt oxide, their oxidation states, and (b) spinel structure.^19^ (B) (a) Representation of biosynthesized cobalt oxide NPs from different biological sources (miscellaneous included gum, sucrose, starch, egg white, cloves, dye powder, walnut skin, endemic species). (b) Number of publications on green synthesis of cobalt oxide NPs in the past eight years using “Green synthesis of cobalt oxide nanoparticles” as cumulative keywords (source: Web of Science) reported till 21st Sep, 2024.

The cost-effectiveness and abundance of cobalt oxide make it an economically viable choice for large-scale production, ensuring widespread accessibility and affordability. Its exceptional chemical stability ensures durability and longevity in various conditions. Cobalt oxide exists in four forms: CoO, CoO_2_, Co_2_O_3_, and Co_3_O_4_,^18^ where cobalt exhibits in (Co^2+^, Co^3+^, and Co^4+^) oxidation states (Fig. 1A) with CoO and Co_3_O_4_ being the most stable. In the spinel structure (AB_2_O_4_), Co^2+^ occupies eight tetrahedral sites (A-sites) and Co^3+^ sixteen octahedral (B-sites). Co_3_O_4_, with its spinel structure, is stable below 891 °C and decomposes to CoO above this temperature. It has a favorable band gap (1.48–2.19 eV), making it useful in supercapacitors.^20–23^ Cobalt oxide NPs are cost-effective, abundant, antiferromagnetic p-type semiconductors with high resistance to oxidation and corrosion.^24–28^ They are used in gas sensing,^29^ antimicrobial and anticancer applications,^30,31^ supercapacitors,^32^ electrocatalysis,^33^ lithium-ion batteries,^34^ energy storage,^35^ water splitting,^36^ dye removal,^24^ CO_2_ reduction,^37^ and drug delivery.^38^

Conventional synthesis methods, such as hydrothermal,^39^ co-precipitation,^40^ sol–gel method,^41^ chemical reduction method,^42^ spray pyrolysis,^43^ chemical vapor deposition (CVD),^44^ microwave-assisted,^45^ solvothermal,^46^ thermal decomposition,^47^ casting and irradiation technique,^48^ auto combustion,^49^ microemulsion,^50^ sonochemical,^51^ laser ablation,^52^ mechanochemical processes,^53^ ionic liquid assisted method,^54^ reflux method,^55^ polyol,^56^ pulsed laser deposition,^57^ template method,^58^ and wet synthesis^59^ are expensive, environmentally hazardous, and time-consuming. For instance, hydrazine hydrate (NH_2_NH_2_), and sodium borohydride (NaBH_4_) were commonly used toxic reducing agents.^60,61^ In contrast, biogenic methods, using plant extracts, microorganisms, algae, and waste materials, are cost-effective, environmentally friendly, and quicker. Green synthesis avoids toxic chemicals and surfactants, leading to biocompatible, stable NPs with low band gap energy due to quantum confinement effects. This makes biogenic cobalt oxide NPs more sustainable and less harmful to the environment.^62–64^

Researchers are increasingly focusing on environmentally friendly methods for fabricating cobalt oxide NPs, with a notable rise in publications on plant-based synthesis, reflecting a growing interest in sustainable approaches (Fig. 1B). The very recent studies on green synthesized cobalt oxide NPs are reported in Table 1. In a recent study, lemon extract was employed for cobalt oxide NP formation for LPG gas sensor application.^29^ Similarly, the utilization of rotten apple juice was investigated for preparing surface-modified cobalt oxide nanostructures for efficient oxygen evolution.^28^ The collective recent studies on the green synthesis of cobalt oxide NPs using various natural extracts demonstrate their potential in antibacterial, anticancer, and antioxidant activities along with supercapacitor performance, photocatalytic, as well as gas sensing applications, highlighting their multifunctionality and efficacy across diverse fields.^62,81,82^

**Table 1:** Recent studies of green synthesized cobalt oxide NPs

Natural source	Precursors	Part	Shape/morphology	Size	Applications	Ref.
*Carica papaya*	Co(NO_3_)_2_·6H_2_O	Leaves	Spherical	22 nm	Anti-oxidant, anti-cancer	65
*Cocos nucifera*	Co(NO_3_)_2_·6H_2_O	Fruit	—	18.44 nm	Photocatalytic and antibacterial	66
*Aloe vera*	Co(NO_3_)_2_·6H_2_O	stem	Rod shape	—	Oxygen evolution reaction	67
*Blumea lacera*	CoCl_2_·7H_2_O	Leaves	Spherical	5–10 nm	Antimicrobial	68
*Punica granatum* L.	CoCl_2_·6H_2_O	Seed oil	Spheroidal	129.6 nm	Antimicrobial and anticancer	69
*Punica granatum* L.	Co(NO_3_)_2_·6H_2_O	Fruit	Nano-spherical with a few nano-rod	17.19 nm	Bimedical	70
*Croton Macrostachyus*	Co(NO_3_)_2_·6H_2_O	Leaves	Spherical and irregular	12.75 nm	Antibacterial activity	71
Carboxymethyl cellulose	Co(NO_3_)_2_·6H_2_O	Sugarcane straw	Spherical	27.2 nm	Biological activities	72
*Ziziphus oenopolia*	CoCl_2_·6H_2_O	Leaves	Irregular	27 nm	Antimicrobial	73
*Lawsonia inermis*	Co(NO_3_)_2_·6H_2_O	Leaves and bark	Rough cubic and spherical	98 nm	Bimedical	74
Spent coffee	Co(NO_3_)_2_·6H_2_O	Seed	Spherical and irregular	29.01 nm	Catalytic and photocatalytic dye degradation	75
*Citrus tangerina*	Co(NO_3_)_2_·6H_2_O	Leaves	Octahedral	90–130 nm	Antimicrobial, antioxidant, and anti-inflammatory	76
*Jasminum mesnyi*	Co(NO_3_)_2_·6H_2_O	Leaves	Spherical	59.9 nm	dye degradation	77
*Spirulina platensis*	CoCl_2_·6H_2_O	Blue-green algae	Octahedral	26.1 nm	Catalytic CO oxidation	78
*Chlorella vulgaris*	CoCl_2_·6H_2_O	Green algae	Nanosheets	16.4 nm	Catalytic CO oxidation	78
*Haematococcus pluvialis*	CoCl_2_·6H_2_O	Green algae	Nanosheets	18.4 nm	Catalytic CO oxidation	78
*Nodosilinea nodulosa*	CoCl_2_·6H_2_O	Algae	Spherical and irregular	41 nm	Therapeutics	79
*Luminescent bacterium Vibrio* sp. VLC	Co(NO_3_)_2_·6H_2_O	Bacteria	Spherical	65–67 nm	Antioxidant, antibacterial, and anticancer	80

Previously published review articles on green-synthesized cobalt oxide NPs have largely overlooked their biomedical applications, focusing instead on other areas such as photocatalytic degradation, *etc.* For instance, a recent review by Imtiyaz *et al.*^83^ did not cover biomedical uses in particular. While Iravani *et al.*^84^ touched on biomedical applications, their review primarily emphasized catalytic activities and was published in 2020. Since then, substantial advancements have been made in the field. Similarly, the review by Mubraiz *et al.*^85^ focused solely on antimicrobial properties, providing a limited scope of biomedical potential. This review seeks to address these gaps by offering a comprehensive overview of the biomedical applications of green-synthesized cobalt oxide nanoparticles. It aims to provide timely insights for researchers seeking sustainable approaches in this field, setting it apart from previous works and aligning with the current surge of interest in eco-friendly nanomaterials for medical use.

This review provides a comprehensive analysis of innovative green synthesis techniques utilizing plant extracts and microbial sources. We highlight the mechanisms and eco-friendly attributes of these methods, which present a significant advancement over traditional, hazardous synthesis processes. We delve into the diverse biomedical functionalities of cobalt oxide NPs, including cytotoxicity, larvicidal, antileishmanial, hemolytic, anticoagulating, thrombolytic, anticancer, and drug sensing abilities. This extensive range of applications showcases the versatility and potential of these NPs in medical science. The review highlights the importance of sustainability in nanomaterial synthesis. By utilizing natural resources and biological agents, we demonstrate how cobalt oxide NPs can be synthesized in a cost-effective, sustainable, and biocompatible manner, addressing the environmental concerns associated with conventional methods. This article provides a prospective analysis on the future of cobalt oxide NPs in biomedicine. By highlighting the gaps in current research and the avenues for further exploration, we aim to inspire researchers to advance sustainable practices in nanotechnology and contribute to the development of innovative solutions for human and environmental well-being.

## 2. Synthesis of green synthesized cobalt oxide NPs

**Fig. 2:**
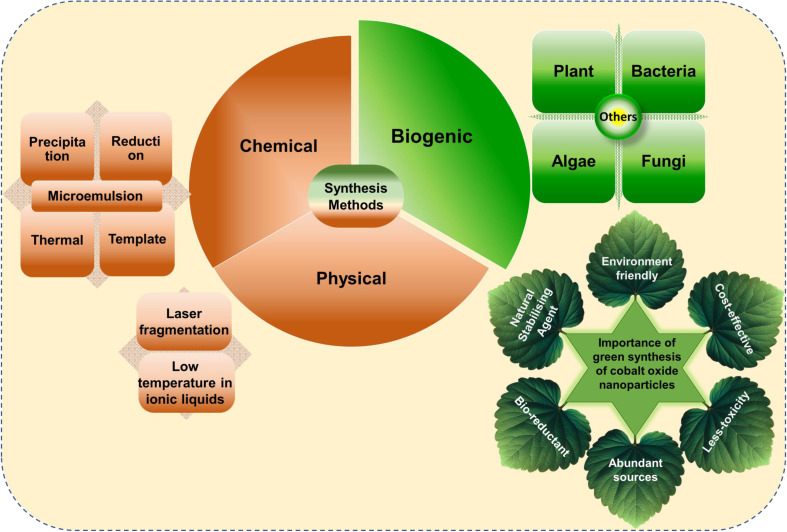
Representation of frequently used conventional and green methods for cobalt oxide NPs synthesis and their importance.

Biogenic synthesis provides an eco-friendly, biocompatible alternative to conventional methods for cobalt oxide NPs production. Plants and microorganisms serve as sustainable sources for NP synthesis, utilizing phytochemicals and biomolecules such as flavonoids, proteins, and enzymes to stabilize NPs, minimizing the need for chemical reagents like stabilizers or reducers. This green approach aligns with principles of green chemistry by avoiding harmful chemical waste.^64^Fig. 2 illustrates the most frequently used synthesis methods and highlighted the importance of green synthesized cobalt oxide NPs. Moreover, plants and microbes offer essential compounds that act as reducing and capping agents, facilitating NP formation. Various shapes, sizes, and properties of cobalt oxide NPs have been achieved through this method.^64,86,87^

### 2.1 Source-based synthesis protocol for green synthesized cobalt oxide NPs

#### 2.1.1 Plant extracts (leaves, roots, seeds, bark, fruits, flowers, rhizomes)

**Fig. 3:**
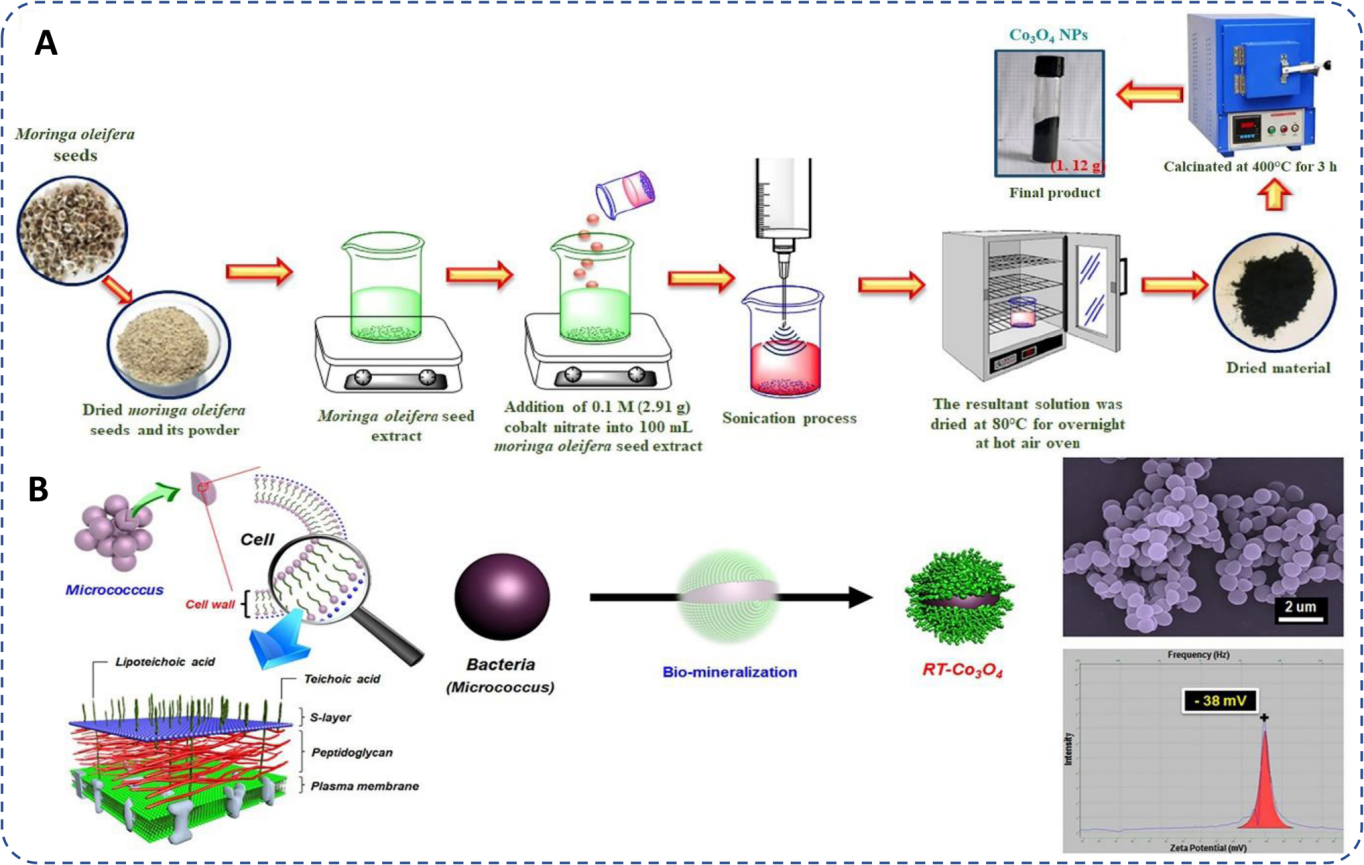
Schematic representation of general protocol of (a) plant sourced (seed)^93^ and (b) microbe (*Micrococcus* bacteria)^94^ based biogenic synthesis of cobalt oxide NPs.

Different plant fragments, such as leaves, fruits, roots, stems, seeds, and flowers, have been effectively used to synthesize cobalt oxide NPs through green chemistry approaches.^19,21,40,88–90^ To prepare plant extracts, these parts are collected, thoroughly cleaned with distilled or deionized water, and either dried and powdered or used directly.^91^ The fragments are then heated in water or alcohol below 60 °C to preserve the phytochemicals, which play a crucial role in NP formation. The extracts, mixed with a cobalt salt solution, facilitate the synthesis of cobalt oxide NPs at different temperatures.^92^ The bioactive phytochemicals in the extract act as natural reducing and stabilizing agents, eliminating the need for additional chemicals during synthesis^87^ (Fig. 3A).

#### 2.1.2 Algae

Algae, as aquatic eukaryotic organisms, are widely used in NP synthesis due to their rich content of secondary metabolites, proteins, carbohydrates, peptides, and pigments, which act as natural nano-biofactories.^82^ These organic compounds help reduce and stabilize the NPs. The algal extract is prepared similarly to plant extracts: washing, drying, and grinding the algae into powder, which is then suspended in distilled water and heated to 60 °C for 4 hours. After cooling and filtering, the extract is mixed with a cobalt salt precursor and stirred at room temperature. For example, *Grateloupia sparsa* algae were used to prepare cobalt oxide NPs by mixing the extract with a cobalt precursor, where a color change from pink to brown indicated the formation of the NPs.^82^

#### 2.1.3 Fungi

The procedure responsible for the creation of cobalt oxide NPs involves enzymatic reduction either in the fungal cell wall or within the fungal cell itself. Fungi offer significant advantages over other microorganisms for cobalt oxide NP synthesis due to their fast growth and higher NP yield.^95^ This is attributed to the presence of reducing agents, intracellular enzymes, and proteins on their cell surfaces. The process begins by adding fungi, either from bread or other sources, to a cobalt salt precursor at 28–30 °C and pH 6.5–11, followed by incubation for 3–4 days. The color change depends on the precursor used, varying from yellow to reddish or olive green. Cobalt oxide NP formation occurs through enzymatic reduction, either on the fungal cell wall or inside the cell itself.^36,85^

#### 2.1.4 Microbes or microorganisms

The microbiologically induced precipitation (MIP) is a promising, clean, and, sustainable, technique compared to conventional methods.^96^ The process starts by developing microbial cultures using nutrient broth, followed by suspending the culture in distilled or deionized water. The precursor solution is then added to the bacterial suspension and stirred at room temperature. The resulting mixture is centrifuged at ∼5000 rpm for 10–20 minutes to collect the NPs, which are washed multiple times with distilled water. Finally, the NPs are dried in a vacuum oven at ∼60 °C for 5–6 hours^94,97^ (Fig. 3B).

### 2.2 Role of bio-reductants in green synthesis of cobalt oxide NPs

**Fig. 4:**
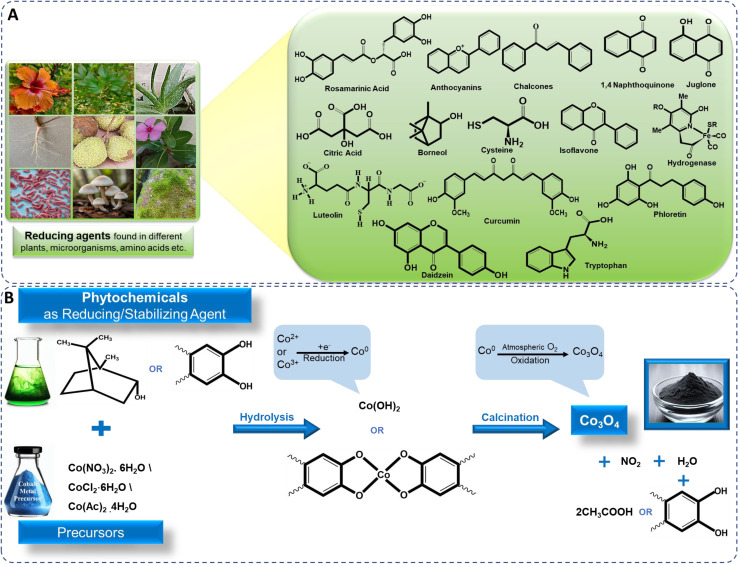
Illustration of (A) bioreduction and (B) mechanistic approach for biosynthesis of cobalt oxide NPs.

Bio-reductants from natural resources, particularly plants, are extensively used to reduce cobalt metal precursors and stabilize cobalt oxide NPs. Plant extracts, rich in biomolecular compounds like flavonoids, terpenoids, alkaloids, and phenols, contain functional groups such as hydroxyl (–OH), carboxyl (–COOH), carbonyl (–C=O), which play crucial roles in NP reduction and stabilization (Fig. 4A). For example, flavonoids chelate with metal ions, reducing them to form NPs, while other biomolecules prevent agglomeration and stabilize NPs, controlling morphology.^89,98^ Various plant extracts like *Azadirachta indica*,^24^*Calotropis gigantea*,^91^*Curcuma longa*,^99^*Punica granatum*,^100^ and microbes like *Aerva javanica*, *Fusarium oxysporum*, *Bacillus subtilis*,^85^*Grateloupia sparsa*,^62^*Aspergillus nidulans*,^101^*etc.* are used in cobalt oxide NP synthesis. Algae contains several secondary metabolites such as proteins, organic molecules, like carbohydrates and polyphenols, polysaccharides, and phytochemicals having –NH_2_, –OH, and –COOH functional groups, also act as reducing agents.^62,82^ Similarly, fungi, containing bioactive compounds and redox enzymes, and bacteria that utilize bioreduction, contribute to cobalt oxide NP formation, offering renewable, non-toxic alternatives to hazardous chemicals.^85^

### 2.3 Mechanism involved in green synthesis of cobalt oxide NPs

Plants produce NPs due to phytochemicals like phenolic acids, flavonoids, tannins, and vitamins, which act as ligands. These biomolecules prevent NP aggregation, regulate morphology, and stabilize the NPs.^23,102^ These phytochemicals have the ability to bind to cobalt ions. Most common precursors used for the synthesis of cobalt oxide NPs are cobalt sulphate, the hydrated salts of cobalt nitrate (Co(NO_3_)_2_·6H_2_O), cobalt chloride (CoCl_2_·6H_2_O), and cobalt acetate (Co(OAc)_2_·4H_2_O).^17^ Single step *in situ* green synthesis procedure involves both growth and nucleation processes. These processes occur through the reduction of cobalt ions into neutral cobalt atoms, which leads the particles to nucleate and stabilises, with the help of biomolecules present in extracts. The phenolic groups contain –OH and –COOH, which allows strong affinity to combine with metals, this affinity is particularly evident when these compounds conjugate with ortho-phenolic hydroxyl groups and ester oxygen atoms.^100^ The hydroxyl aromatic ring groups of the component of the extract react with a cobalt precursor, undergoes hydrolysis due to the presence of hydroxyl groups in the aromatic portion of the biomolecules, leading to the formation of a complex-ligand with cobalt ions or triggering the formation of hydroxide (Co(OH)_2_). This includes electron donation by the phytochemical compound to cobalt ions (Co^2+^ or Co^3+^) derived from cobalt precursor compound, leading to the reduction of cobalt ions to cobalt metal atoms on the surface of NPs,^100^ (Fig. 4B), depicted in eqn (i) and (ii).

i Co^2+^ + 2bioreducant-OH → Co + bioreducant-O + 2H^+^

ii Co(NO_3_)_2_ + polyphenol-OH → Co + oxidized polyphenol-O + HNO_3_

The resulting Co(OH)_2_ and complex-ligand with cobalt ions are subsequently subjected to calcination, leading to its decomposition and the release of water, ultimately yielding cobalt oxide NPs. The cobalt metal atoms aggregate to form small nuclei and simultaneously, oxygen molecules either from the reaction environment or atmosphere react with cobalt metal atoms to form cobalt oxide. This continuous process on the surface of cobalt metal nuclei to form cobalt oxide NPs,^24,103^ depicted in eqn (iii) and (iv).

iii 6Co + 4O_2_ → Co_3_O_4_

iv 3Co^o^ + 2/3O_2_ → Co_3_O_4_

The additional phytochemical components present in the plant extracts are also serve as capping or stabilizing agents for the synthesized cobalt hydroxide before the calcination process. The chemical reactions including nucleation and shaping, contribute to the generation of stabilized NPs preventing agglomeration, depicted in eqn (v).

v Co_3_O_4_ + 2bioreducant-OH (ligand) → capped Co_3_O_4_

However, in the literature, there are no direct studies conducted on how cobalt oxide precursors affect the reactivity of these plant-based reagents. The actual mechanism is still unknown but the cobalt oxide precursors have the potential to engage in complexation with phytochemicals, that act as reducing and stabilizing agents thereby inducing chemical modifications that are influenced by pH and temperature parameters.^83^ They affect the solubility and dispersion characteristics, ultimately impacting the stability and degradation rate of plant-based reagents which influences their reactivity.^1^ Thus, by these factors cobalt oxide precursors affect the reactivity of plant-based reagents and optimized green synthesis process.

## 3. Characteristics of green synthesized cobalt oxide NPs

The fundamental characteristics of biogenically produced cobalt oxide NPs such as shape, size, crystallinity and stability are the important criteria in determining their application.

### 3.1 Visual observation, UV-visible and FTIR spectroscopic analysis

**Fig. 5:**
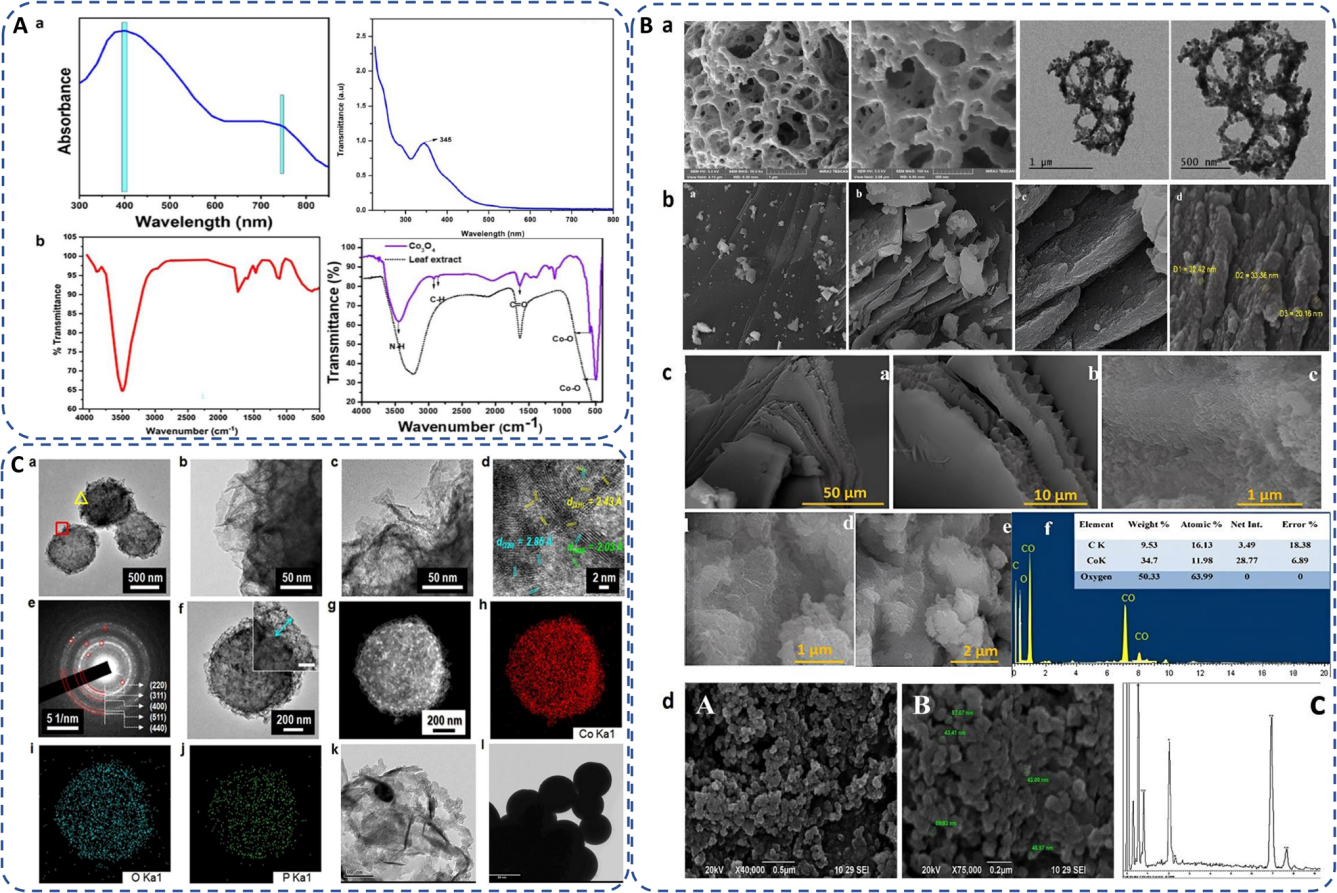
(A) (a) UV-Vis spectra of green synthesized cobalt oxide NPs using red marine algae extract (*Grateloupia sparsa*)^62^ and *Curcuma longa* root extract.^105^ (b) FTIR spectra of green synthesized cobalt oxide NPs using red marine algae extract (*Grateloupia sparsa*)^62^ and *Mollugo oppositifolia* leaf extract.^21^ (B) SEM images of biogenically synthesized cobalt oxide NPs employed with (a) *Hyphaene thebaica* fruit extract,^88^ (b) *Rosmarinus officinalis* leaf extract,^19^ (c) *Grateloupia sparsa* marine algae extract (with EDX)^62^ and (d) *Microbacterium* sp. MRS-1 metal resistant bacteria (with EDX).^106^ (C) (a–d) TEM and image of the microbe mediated Co_3_O_4_ NPs, (e) SAED pattern of porous-Co_3_O_4_/bacteria, (f) TEM image of single Co_3_O_4_ NPs, (g–j) HAADF STEM image of cobalt oxide NPs^94^ and (k and l) TEM images of sub-spherical cobalt oxide NPs prepared by rosemary leaf.^20^

Synthesis of cobalt oxide NPs can be predicted by analyzing the color change of the reaction mixture,^104^ or by analyzing UV-Vis-spectroscopic data, which shows significant peaks of cobalt oxide NPs in the range of 340 to 500 nm (Fig. 5A(a)).^105^ The green synthesized powdered cobalt oxide NPs are visually dark olive greenish in color. The peak around 400 nm is ascribed to surface plasmon resonance behaviour.^62^ Synthesized NPs should be centrifuged at high rpm to isolate cobalt oxide NPs followed by washing multiple times and drying at a mild temperature (∼50 °C).^107^ After drying and/or further calcination at around 400 °C, the NPs turned to black powder. Contrarily, in case of bacteria, for instance, *Microbacterium* sp. MRS-1, the color of nutrient broth changes from light pink to dark pink as initial confirmation of formation of extracellular cobalt oxide NPs.^106^

Cobalt oxide NPs synthesized by *Mollugo oppositifolia* leaf extract shows a broad peak at 3465.93 cm^−1^ suggesting the presence of the N–H group of an amine moiety, C–H functional group alkanes signal appeared between 2800 and 3000 cm^−1^. The presence of both tetrahedral and octahedral Co–O vibrations can be verified by the peaks at 509.59 cm^−1^ and 584.80 cm^−1^, respectively (Fig. 5A(b)),^21^ showing the presence of surface ligands or organic biomolecules by detecting the shifting in characteristic absorption bands associated with metal–oxygen stretching vibrations. FTIR spectra of Co_3_O_4_ NPs prepared by red algae extract, exhibit a broad peak at about 3500 cm^−1^ indicating the presence of amide, primary and secondary amine groups, polyphenol, and alcohol groups due to OH–NH bending. The presence of aromatic rings in plant structures, the bending of –OH and C–O stretching in alcohols and carboxylic acids are indicated by peaks at 1669, 1413, and 1079 cm^−1^, suggesting their contribution to the formation of cobalt oxide NPs. Furthermore, the peaks at 760 and 562 cm^−1^ in the spectrum of Co_3_O_4_ NPs are connected to Co^2+^ and Co^3+^ vibrations in a tetrahedral hole in the spinal lattice (Fig. 5A(b)).^62^ If not calcinated, FTIR spectra exhibit additional peaks in green synthesis due to organic functional groups from the used biological source as compared to the chemical synthesis.^83,108^

### 3.2 Morphology

Green synthesis methods often involve the use of natural bioactive compounds or biomolecules that can functionalize the surface of NPs. SEM provides detailed images of the surface morphology of cobalt oxide NPs and assess their shape, uniformity, coverage, and aggregation behaviour, providing insights into the interaction between cobalt oxide NPs and their immediate environment to understand how green synthesis methods affect the physical properties of cobalt oxide NPs as well as provide optimization synthesis parameters to control their morphology for their reproducibility and consistency in various applications.^24,109^

The morphology of biosynthesized cobalt oxide NPs are studied using FE-SEM and TEM. For instance, Shim *et al.*^97^ synthesized *Bacillus subtilis*-directed porous Co_3_O_4_ nanorods and most of the biosynthesized nanorods had a closed-end, but few of them were open-ended type. Fig. 5(B and C) depicts different types of morphologies observed by SEM and TEM analysis of the green synthesized Co_3_O_4_. Safdar *et al.* revealed porous structure of *Hyphaene thebaica* fruit extract mediated synthesized Co_3_O_4_ NPs.^88^ Semi-triangular pyramidal shape of *rosemary leaf* extract biosynthesized Co_3_O_4_ NPs was confirmed by TEM images.^20^ Sheet-like morphology was found in *Rosmarinus officinalis* mediated CoO/Co_3_O_4_ NPs.^19^ Similarly, cobalt oxide NPs synthesized using red algae extract also exhibit complicated sheet-like structures.^62^*Microbacterium* sp. MRS-1 is a heavy metal resistant bacterium. Sundararaju *et al.* isolated the bacteria from the electroplating industrial effluent to reduce cobalt metal to cobalt oxide NPs and thus detoxification of metal ions from the wastewater. The agglomerated Co_3_O_4_ NPs were spherical and pentagon accompanied with 54 : 34 Co : O elemental ratio in the range of 10–70 nm, observed by SEM-EDX (Fig. 5B(d)).^106^Tables 2–6 displays the various morphologies and size range of green synthesized cobalt oxide NPs using different source as reducing or stabilizing agent and different precursors along with their applications.

### 3.3 Size

The size of biogenically produced cobalt oxide NPs are generally analyzed by DLS study, TEM imaging and XRD of the NPs. The average crystallite size of cobalt oxide NPs can be determined by XRD peaks and calculated by using the Debye–Scherrer eqn (1) (ref. 21) as follows:1
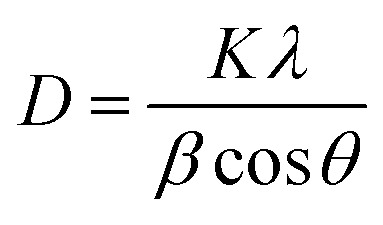
where *D* is the average crystallite size, *K* is the shape factor (0.9), *λ* is the wavelength of X-ray radiation, *β* is the full width at half maxima and *θ* is the Bragg's angle.

Raimundo *et al.*^110^ reported that cobalt oxide NPs synthesized using *Rhodophyta* showed an average size of 65 nm. Small sized 2–5 nm and 6.7 nm nanocrystals were produced using *Ipomoea carnea* leaf extract^104^ and *Calotropis procera* latex,^111^ respectively. *Aerva javanic*a (plant), *Bacillus subtilis* (bacterial strain), and *Fusarium oxysporum* (fungus) derived cobalt oxide NPs revealed particle sizes of 39.23 nm, 31.2 nm and 33.4 nm respectively.^85^ High annealing temperature at 800 °C shows rapid increase in size to 39.44–53.56 nm for Arista leaves extract synthesized NPs.^87^ Shim *et al.* prepared *Micrococcus lylae* bacteria-mediated Co_3_O_4_ NPs had a crystalline size of 2–10 nm, forming mesoporous structures (Fig. 5C(a–j)).^94^ The smaller size of Co_3_O_4_ NPs allows them to penetrate bacterial membranes more easily. Smaller nanoparticles exhibit better dispersion and reduced aggregation, which ensures uniform distribution and more efficient antimicrobial action. After penetration, they can disrupt internal cellular processes, such as enzyme functions and DNA replication, enhancing their bactericidal properties.

**Fig. 6:**
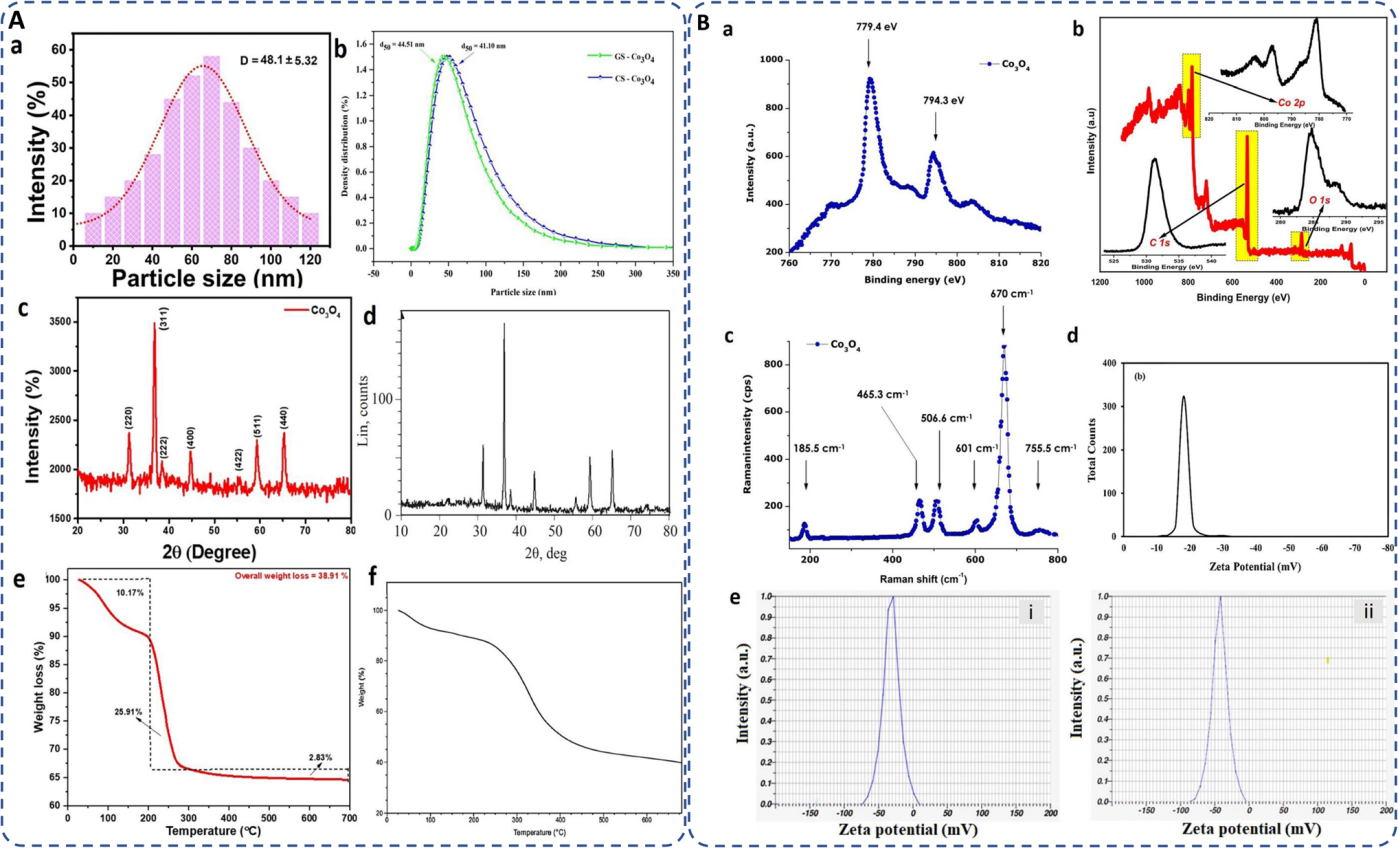
(A) (a and b) DLS analysis for particle size distribution of biosynthesized cobalt oxide NPs by utilizing marine red algae^62^ and *Moringa* seed extract;^93^ (c and d) XRD pattern of cobalt oxide NPs synthesized by *Mollugo oppositifolia* L. leaf extract^21^ and gelatin;^115^ (e and f) TG curve of *Muntingia calabura*^116^ and bacteria mediated (*Microbacterium* sp. MRS-1) biosynthesized cobalt oxide NPs.^106^ (B) (a and b) XPS of *Aspalathus linearis* plant extracNt^117^ and *Muntingia calabura* leaf extract^116^ mediated green synthesized cobalt oxide NPs; (c) Raman spectra of biosynthesized annealed cobalt oxide NPs;^117^ (d and e) zeta potential of *Rosemary* extract,^20^ and (e) lemon extract^29^ mediated green synthesized cobalt oxide NPs.

The hydrodynamic diameter,^6,112,113^ crucial for stability and dispersibility, was measured as 218 nm for Akhlaghi *et al.*^114^ Co_3_O_4_ NPs, larger than the SEM size of 6–20 nm, likely due to the presence water molecules layers on the surface of cobalt oxide NPs.^20^*Geranium wallichianum*-derived NPs showed an aggregate size of 320 ± 2.33 nm in DLS analysis,^26^*Ipomoea carnea* NPs measured 4–10 nm (mostly 7.83 nm).^104^ DLS analysis of red algae-synthesized NPs showed 48.1 ± 5.32 nm (Fig. 6A(a)).^62^ The *Moringa* seed extract mediated cobalt oxide NPs had an average size of 44.51 nm compared to 41.10 nm for chemically synthesized NPs (Fig. 6A(b)).^93^

Microbial *Bacillus subtilis*, *Fusarium oxysporum*, mediated cobalt oxide NPs exhibit 30.9191°, 36.5364°, 59.0089°, and 64.9460° diffraction peaks for the corresponding planes (220), (311), (511), and (440) and with average crystallite size 31.2 nm and 33.4 nm, respectively.^85^ Gowthami *et al.* reported a pure FCC spinel structure of cobalt oxide NPs using *Mollugo oppositifolia* L. leaf extract. Diffraction peaks at 31.2°, 37.6°, 38.7°, 44.8°, 55.6°, 59.8°, and 66.3° correspond to *hkl* values (220), (311), (222), (400), (422), (511), and (440) (Fig. 6A(d)).^21^ XRD pattern of highly pure cobalt oxide (CoO) NPs prepared by gelatin, as depicted in Fig. 6A(c), are observed in 2*θ* range of 10–80°, with average particle size of about 25 nm.^115^ Since, phytochemicals influence the nucleation and growth process to favor the fcc structure, overall, the fcc structural arrangement of cobalt oxide maximizes the density, stability and minimizes the repulsive interactions, *i.e.* high packing efficiency of the spinel structure by occupying interstitial sites within the oxygen lattice. However, the XRD peaks are often display sharper peak in case of chemical synthesis while broader peak in green synthesis indicating less crystallinity but contrarily minimizing their toxicity.

### 3.4 Surface interface: composition and chemistry

Surface of the NP plays a vital role in determining its applicability. Surface area of bio-inspired cobalt oxide NPs are estimated using Brunauer–Emmett–Teller (BET) techniques. For instance, the BET surface area of cobalt oxide NPs synthesized by the *Calotropis gigantea* plant was found 46.7 m^2^ g^−1^. Generally, large surface area of NPs results in a significant increase in the number of active sites and a greater number of adsorbed substrates for the intended catalytic reaction.^91^ Recently, rice husk derived silica gel was used in synthesizing cobalt oxide NPs possessing 735 m^2^ g^−1^ surface area which were more than the Co_3_O_4_/MCM-41 nanocomposites, have a surface area of 608 m^2^ g^−1^. This increase in surface area leads to more exposed catalytic sites for light irradiation, and higher levels of acephate molecules adsorbed.^118^ It should be noted here that since the synthesis method and phytochemicals affect the size and dispersion of the NPs, they consequently influence the surface area of the cobalt oxide NPs. Smaller particles exhibit a high surface-to-volume ratio, resulting in a higher surface area, which enhances their utility in adsorption, catalysis, sensing, conductivity, and therapeutic applications. This high surface area allows for better interaction with cells and tissues, improves solubility, and enhances catalytic activity, making them more effective in therapeutic and diagnostic applications. However, the optimal surface area also depends on the specific biomedical application, with smaller NPs being more suitable for drug delivery, while slightly larger ones may be better for imaging or diagnostics. There are examples which shows varying surface area as per aforementioned reasons, such as *Bacillus subtilis*-directed porous cobalt oxide NPs observed surface area of bacteria and cobalt oxide NPs at 5.1 and 73.3 m^2^ g^−1^, respectively, but the thermal treatment enhanced the bacteria/cobalt oxide hybrid nanorod surface area to 11.2 m^2^ g^−1^.^97^l-Cysteine,^119^*Grateloupia sparsa*,^62^ and *Terminalia chebula*^120^ mediated Co_3_O_4_ NPs found 24.6 m^2^ g^−1^, 35.21 m^2^ g^−1^, and 22 m^2^ g^−1^ surface area, respectively.

Additionally, XPS and Raman spectroscopy identify the elemental composition and oxidation states of cobalt and characterize the vibrational modes or chemical bonds associated with the crystal lattice present on the NP surface. For instance, *Aspalathus lineari* plant extract mediated cobalt oxide NPs shows the characteristic XPS peak of Co_2p_ with O_1s_ consistency and attributed binding energies of 2p_1/2_ and 2p_3/2_ at 779.4 and 794.3 eV, respectively (Fig. 6B(a)).^117^ Similarly, Vinayagam *et al.* reported two compatible peaks for Co^3+^ and Co^2+^ oxidation states of cobalt oxide NPs attributed to the binding energies at 797.13 (Co 2p_1/2_) and 781.02 (Co 2p_3/2_) eV accompanied by a 16.11 eV peak separation. For O 1s, a broad peak at 531.28 eV indicates its binding with both the oxidation states of cobalt. Furthermore, the organic components from the extract are involved by the presence of a C 1s peak at 284.81 eV (Fig. 6B(b)).^116^

Raman spectroscopy is sensitive to the presence of metal–oxygen bonds and can differentiate between different cobalt oxide phases (CoO and Co_3_O_4_). Diallo *et al.* reported tetrahedral (Co^2+^ (3d^7^)) and octahedral sites (Co^3+^ (3d^6^)) of biosynthesized cobalt oxide NPs, corresponds to the active modes of Co_3_O_4_ spinel, *Fd*3*m* symmetry as: Γ = A_1g_ (R) + E_g_ (R) + F_1g_ (IN) + 3F_2g_ (R) + 2A_2u_ (IN) + 2E_u_ (IN) + 4F_1u_ (IR) + 2F_2u_ (IN), where (IN), (IR), and (R) are the inactive, infrared-active, and Raman active vibrational modes, respectively. The characteristic Raman vibrational modes are present at ∼185.5, ∼465.3, ∼506.6, ∼601, ∼670, and ∼755.5 cm^−1^, with a combined octahedral site A1_g_, the E_g_, and F2_g_ modes combined with tetrahedral site with an average shift of Δ*ν* ∼ 5 cm^−1^ due to stress strain surface ratio (Fig. 6B(c)).^117^

### 3.5 Stability

The thermal and colloidal stability of biogenically synthesized cobalt oxide NPs are one of the important parameters that can be assessed using TGA and zeta potential. For instance, *Muntingia calabura* leaf-derived NPs exhibited a total weight loss of 38.91% between 28–300 °C due to moisture loss and organic breakdown, followed by a 2.83% loss at 300 °C indicating CoO to Co_3_O_4_ phase transformation (Fig. 6A(e)).^116^ Similarly, gelatin-mediated cobalt oxide nanocrystals displayed water loss at 50–110 °C, organic combustion at 350 °C, and pure cobalt oxide formation between 350-460 °C, with no weight loss beyond 460 °C, confirming stability.^115^*Grateloupia sparsa* algae-derived NPs, lost 6.3% at 260 °C and 17.6% at 410 °C indicating gradual organic breakdown^82^ and *Microbacterium* sp. MRS-1 bacteria derived NPs displayed mass losses of 7%, 15%, and 50% at 100 °C, 225 °C, and 250–400 °C, respectively, remaining stable up to 700 °C (Fig. 6A(f)).^106^ For colloidal stability, *Trigonella foenumgraceum*-derived NPs^114^ had a negative zeta potential of −20.4 mV, indicative of surface hydroxyl groups, while *Rosemary* leaf-based NPs exhibited −18 mV (Fig. 6B(d))^20^, ensuring good colloidal stability. NPs synthesized from *Geranium wallichianum*^26^ and *Cordia myxa* demonstrated zeta potentials of −10.5 mV and −40 mV, respectively, indicating phytochemicals' influence on surface charge, promoting dispersion and preventing aggregation.^92^ Similarly, *Bacillus subtilis* mediated porous NPs presented −46 mV, due to peptidoglycan presence.^97^ Lemon extract derived NPs showed even higher zeta potentials of −33.4 mV (unheated) and −42.1 mV (heated), ensuring strong repulsion and dispersion stability (Fig. 6B(e)).^29^ Higher zeta potential values increase electrostatic repulsion, enhance stability and reduce aggregation in the medium. Overall, the functional groups of biomolecules, acting as natural stabilizers, on the surface of cobalt oxide NPs impart a surface charge that directly influences the zeta potential and hence enhanced their stability as compared to chemically synthesized NPs.

## 4. Green synthesized cobalt oxide NPs: literature analysis

### 4.1 Using plant source

Nowadays, various plant sourced extracts have been employed in the green synthesis of cobalt oxide NPs, unveiling diverse applications, such as leaves, roots, fruits, peel, and bark, are used very often as capping, and reducing agents.^93^ For instance, *Cirsium vulgare* leaf extract produced NPs averaging 20 nm, exhibiting catalytic capabilities in l-cysteine oxidation.^121^*Sechium edule* fruit extract facilitated the synthesis of monocrystalline spinal cobalt oxide NPs through biochemical reduction of CoOOH to Co(OH)_2_, achieving high reproducibility in H_2_O_2_ detection.^122^*Ipomoea carnea* extract yielded cobalt oxide NPs with a diminutive size of 6.78 nm, demonstrating robust antibacterial activity against *Staphylococcus aureus* and *Shigella flexneri*, with inhibitory zones of 17.8 nm and 16.7 nm, respectively, comparable to vancomycin and ampicillin antibiotics.^104^ Bronzato *et al.* utilized tomato seed extract to synthesize cobalt oxide quantum dots (QDs) sized at 4.5 nm, effective in Ciprofloxacin degradation.^123^ Rosemary leaf extract, rich in carnosol, carnosic acid, rosmanol, isorosmanol, and caffeic acid, synthesized cobalt oxide NPs that displayed potent antioxidant properties and demonstrated efficacy as anticancer agents against Gep G2 and MCF7 cancer cells.^20^ There are a wide variety of plant source available to synthesize cobalt oxide NPs. A list of different plant source and their applications are presented in Table 2.

**Table 2:** Plant-based green synthesized cobalt oxide NPs

Natural source	Precursors	Part	Shape/morphology	Size	Applications	Ref.
*Mollugo oppositifolia*	CoCl_2_·6H_2_O	Leaves	Spherical	22.7 nm	Antimicrobial activity	21
*Geranium wallichianum*	Co(OAc)_2_·4H_2_O	Leaves	—	21 nm	Enzyme inhibition, antioxidant, cytotoxic and antimicrobial	26
Palm kernel oil	CoCl_2_·6H_2_O and Co (NO_3_)_2_·6H_2_O	Seed	Diamond, hexagonal	9–22 nm		40
*Phytolacca dodecandra*	Co(NO_3_)_2_·6H_2_O	Leaves	Spherical	10.79 nm	Antimicrobial activity	63
*Rosmarinus officinalis* (*rosemary*)	CoCl_2_·6H_2_O	Leaves	Sheets like	19–33 nm	Anticancer activity	19
*Arishta*	CoCl_2_·6H_2_O	Leaves	—	40–50 nm	Antibacterial, antifungal activity	87
*Ziziphus oxyphylla* (beri)	CoCl_2_·6H_2_O	Root	Irregular and spherical	40–60 nm	Antibacterial activity	89
*Hibiscus rosa sinensis*	C_4_H_6_CoO_4_	Leaves	Spherical or elliptical	18.98 ± 8.45 nm	Biomedical applications	90
*Cordia myxa*	Co(NO_3_)_2_·6H_2_O	Leaves, roots, and fruit	Prism	47–48 nm	Biological and photocatalytic activity	92
*Aerva javanica*	CoCl_2_·6H_2_O	Aerial	Poly shaped	39.23 nm (methanolic extract) 68.9 nm (aq. Extract)	Antimicrobial activity	85
*Curcuma longa*	Co(NO_3_)_2_·6H_2_O	Leaves	Spherical	22 nm	Photocatalytic and antibacterial	99
*Litchi chinensis*	Co(OAc)_2_·4H_2_O	Fruit	Spherical	26–40 nm	—	102
*Ipomoea carnea*	CoCl_2_·6H_2_O	Leaves	Spherical	6–10 nm	Micronutrient and antimicrobial activity	104
*Curcuma longa*	Co(NO_3_)_2_·6H_2_O	Root	Spherical	97.5 ± 35.1 nm	Photocatalytic dye degradation, antimicrobial and anticancer activity	105
*Trigonella foenumgraceum* (Fenugreek)	CoCl_2_·6H_2_O	Leaves	Spherical	13.2 nm	—	114
*Calotropis procera*	Co(OAc)_2_·4H_2_O	Latex	Spherical	10 nm	Antibacterial activity	111
*Aspalathus linearis*	Co(NO_3_)_2_·6H_2_O	Leaves	Quasi-spherical	∼3.6 nm	—	117
*Sageretia thea* (Osbeck)	Co(OAc)_2_·4H_2_O	Leaves	Cubic	20.03 nm	Biological applications	124
*Clitoria ternatea*	CoCl_3_·6H_2_O	Flower	Spherical	13–17 nm	Biological activities	125
*Manihot esculenta Crantz* (cassava)	CoCl_2_	Root	Prism like-anchored octahedron	36 nm	—	126
*Aerva lanata*	Co(NO_3_)_2_·6H_2_O	Leaves	Irregular, cubic, hexagonal, and spherical	36.24 nm	Microbial and antioxidant activity	127
*Populus ciliata plant* (safaida)	Co(NO_3_)_2_·6H_2_O	Leaves	Square	15–35 nm	Antibacterial activity	128
*Caccinia macranthera*	Co(NO_3_)_2_·6H_2_O	Seed	Granular shape	30–45 nm	Cytotoxicity and photocatalytic activity	129
*Sesbania sesban*	Co(NO_3_)_2_·6H_2_O	Plant	Spherical	15–30 nm	Antibacterial, antioxidant activity	130
*Psidium guajava*	Co(NO_3_)_2_·6H_2_O	Leaves	Spherical	26–40 nm	Photocatalytic and biological activities	131
*Momordica charantia*	CoCl_2_·6H_2_O	Aerial	Irregular	40–90 nm	Photocatalytic activity	132
*Solanum lycopersicum* (tomato)	Co(NO_3_)_2_·6H_2_O	Seed	Quantum dot	5 nm	Degradation of ciprofloxacin	133
*Piper nigrum*	CoCl_2_·6H_2_O	Seeds	Triangular-like with irregular spherical	40–60 nm	—	134
*Salvia hispanica*	Co(NO_3_)_2_·6H_2_O	Leaves	Spherical	9.218 ± 0.93 nm	Biomedical and photocatalytic	135
*Rosemary*	CoCl_2_·6H_2_O	Leaves	Spherical	50–100 nm	Anticancer activity	136
*Ocimum tenuiflorum* (tulsi)	Co(NO_3_)_2_·6H_2_O	Leaves	Rod		Antifungal activity and sensing	137
*Phoenix dactylifera*	—	Seed	Spherical	∼80 nm	Photocatalytic and antimicrobial	138
*Citrus limon*	CoCl_2_·6H_2_O	Fruit juice	—	—	Antimicrobial activity	139
*Manilkara zapota* (chikoo)	Co(NO_3_)_2_·6H_2_O	Leaves	—	20.23 nm	Antifungal activity	140
*Apium graveolens*	Co(NO_3_)_2_·6H_2_O	Leaves	Irregular	21–55 nm	Antibacterial activity	141
*Camellia sinensis*	Co(NO_3_)_2_·6H_2_O	Stalks	Irregular	21–72 nm	Antibacterial activity	141
*Rosmarinus officinalis*	Co (OAc)_2_·4H_2_O	Leaves	—	∼6.5 nm	Biomedical and photocatalytic application	142
*Senna auriculata*	Co(NO_3_)_2_·6H_2_O	Flower	—	31.94 nm	Antibacterial and antifungal	143
*Rhamnus virgata*	Co(OAc)_2_·4H_2_O	Leaves	—	∼17 nm	Biological applications	144
*Spirulina platensis*	CoCl_2_·6H_2_O	Plant	—	13.28 nm	Antifungal activity	145
*Juglans regia*	CoCl_2_·6H_2_O	Bark	Spherical	—	Environmental, antibacterial, and cytotoxic potential	146
*Withania coagulans*	CoCl_2_·6H_2_O	Plant	Bead, cube	49–59 nm	Antibiotic, antifungal and biofilm activity	147
*Vitis vinifera*	Co(NO_3_)_2_·6H_2_O	Seed	Nanorod	10–20 nm	Catalytic, photocatalytic, and antibacterial activities	148
*Gingko*	Co(CH_3_COO)_2_	Leaves	Irregular	30–100 nm	Electrochemical biosensing	149
*Mappia foetida*	CoCl_2_	Leaves	Spherical	—	Antimicrobial potential	150
*Abies pindrow*	C_4_H_6_CoO_4_·4H_2_O	Leaves	Spherical	17 nm	dye degradation	151
*Euphorbia tirucalli*	CoSO_4_·7H_2_O	stem	Irregular and spherical	1 μm- 100 nm	Breast cancer inhibition	152
*Duranta repens*	(CH_3_COO)_2_Co·4H_2_O	Leaves	—	∼23 nm	Electrochemical analysis of tramadol	153
*Hibiscus rosa sinensis*	Co_3_O_4_	Flower	Tubular like	34.9 nm	Antibacterial activity	154
*Spinacia Oleracea* (Spinach)	CoCl_2_·6H_2_O	Leaves	Agglomerate and spherical	—	—	155
*Punica granatum*	Co(NO_3_)_2_·6H_2_O	Seed	Quasi-spherical	3.5 nm	Photodegradation, catalytic hydrogenation, and antibacterial applications	156
*Lawsonia inermis*	Co(NO_3_)_2_·6H_2_O	Leaves, bark	Cubic and spherical	98.05 nm.	Biological activity	74

Overall, different plants have varying capabilities to stabilize and reduce cobalt ions due to their unique biochemical compositions, which can lead to differences in NP size and shape. Besides, the method of extracting these phytochemicals (*e.g.*, boiling, soaking, or direct extraction) affects their concentration and efficacy in NP synthesis. Parameters such as pH, temperature, reaction time, and the concentration of plant extract and metal salts play critical roles in determining the size, shape, and morphology of the synthesized NPs. Optimization of these factors and selection of plant extracts, while avoiding harmful surfactants, are crucial for NP control. Despite challenges in precise control, biogenically synthesized cobalt oxide NPs offer sustainable solutions with diverse applications, warranting further research.

### 4.2 Using algae

The utilization of algae in green synthesis is gaining attention as it possesses a rapid growth rate, facilitate convenient harvesting, and offer a cost-effective means of scaling up, rendering them highly suitable for the biological synthesis of NPs. For instance, cobalt oxide NPs were synthesized by a one-pot hydrothermal technique using *Grateloupia sparsa* (marine red algae extract) for supercapacitor application.^62^ These red algae extract contain carbohydrates, fats, proteins antioxidants, and pigments like chlorophylls and phycobilins which act as reducing agents. Raimundo *et al.*^157^ reported the synthesis of Co_3_O_4_–CoO nanocomposite by using agar–agar (*Rhodophyta*) in two steps including proteic sol–gel and hydrothermal method, and revealed excellent electrocatalytic performance for OER applications. Their most recent investigations have been reported in Table 3.

**Table 3:** Algae-based green synthesized cobalt oxide NPs

Natural source	Precursors	Part	Morphology	Size	Application	Ref.
*Grateloupia sparsa*	Co(NO_3_)_2_·6H_2_O	Algae	Spherical	28.8–7.6 nm	Hemolytic, antioxidant, anticancer, and antibacterial activities	82
Agar–agar (*Rhodophyta*)	Co(NO_3_)_2_·6H_2_O	Algae	Spherical	19 nm	OER	110

### 4.3 Using fungi

The cobalt oxide NPs synthesized by using fungi due to their manifestation of tolerance and metal bioaccumulation capability, as well as their high binding capacity and intracellular intake.^158^ The process is highly effective in generating NPs with well-defined morphologies (Table 4). For instance, *Aspergillus brasiliensis* fungus was used for the green fabrication of quasi-spherical shaped cobalt oxide NPs by adding fungus in cobalt sulphate under shaking conditions for 72 hours at pH 11 and 30 °C temperature, with an average particles size of 20–27 nm for antimicrobial applications and shows good magnetic properties.^159^

**Table 4:** Fungi-based green synthesized cobalt oxide NPs

Natural source	Precursors	Part	Morphology	Size	Application	Ref.
*Bread fungus*	Co(NO_3_)_2_·6H_2_O	Fungus	Cubic	14–19 nm	Photocatalyst for water splitting	36
*Fusarium oxysporum*	CoCl_2_·6H_2_O	Fungus	Less cubic	33.4 nm	Antimicrobial	85
*Aspergillus nidulans*	Cobalt(ii) acetylacetonate	Fungus	Spherical	20.09 nm	—	101
*Aspergillus terreus*	CoSO_4_·7H_2_O	Fungus	Various shapes	—	—	107
*Aspergillus brasiliensis*	CoSO_4_·7H_2_O	Fungi	Quasi-spherical	20–27 nm	Antimicrobial	159
Yeast	CoCl_2_·6H_2_O	Fungi	Hollow spheres	24 nm	—	160

### 4.4 Using microbes or bacteria

Bacterial species have been widely used for the production of NPs because of their high yield, easy handling, and low purification cost.^161^ Bacteria can reduce metal ions present in the metal precursors during the formation of NPs.^95^Table 5 shows several investigations reported the utilization of bacteria in the production of cobalt oxide NPs. Under ambient conditions, porous Co_3_O_4_ hollow rod-shaped cobalt oxide NPs were synthesized through the electrostatic interaction between the functional surface structures of *B. subtilis* bacteria and cobalt ions, exhibits exceptional electrochemical properties when utilized as electrode materials in rechargeable Li-ion batteries.^97^ The biosynthesized spherical cobalt oxide NPs were prepared by adding the bacterial biomass of *Microbacterium* sp. MRS-1 to the cobalt chloride solution. The reduction occurred outside the cell enables the straightforward retrieval of NPs without causing harm to cells, while simultaneously detoxifying toxic metal ions.^106^ Similarly, a 3D hierarchical, flower-like microsphere and porous-Co_3_O_4_ superstructures were facilitated by *Micrococcus lylae*, a Gram-positive bacterium for supercapacitor applications.^165^Table 6 describes other different natural sources that are used in biogenic production of cobalt oxide NPs such as walnut, starch, onion, egg white, sucrose, *etc.*

**Table 5:** Bacteria-based green synthesized cobalt oxide NPs

Natural source	Precursors	Part	Morphology	Size	Application	Ref.
*Micrococcus lylae*	CoCl_2_·6H_2_O	Bacteria	Flower-like	2–10 nm	Supercapacitor	94
*Bacillus subtilis*	CoCl_2_·6H_2_O	Bacterial strains	Rod-like	31.2 nm	Antimicrobial	85
*Bacillus pasteurii*	Co(NO_3_)_2_·6H_2_O	Bacteria	Non-specific	10–31 nm	Supercapacitor	96
*Microbacterium* sp.	CoCl_2_	Bacteria	Spherical	10–70 nm	—	106
*Bacillus subtilis*	CoCl_2_·6H_2_O	Bacteria	Rod	3–5 nm	—	162
*Brevibacterium casei*	Co(OAc)_2_·4H_2_O	Bacteria	Quasi-spherical	5–7 nm	—	163
*Bacillus subtilis*	CoCl_2_·6H_2_O	Bacteria	Hollow rod	2–5 nm	Lithium storage	164

**Table 6:** Other biosource-based green synthesized cobalt oxide NPs

Natural source	Precursors	Part	Morphology	Application	Size	Ref.
*Araucaria heterophylla*	Co(NO_3_)_2_·6H_2_O	Plant gum	Spherical	Catalytic degradation, antimicrobial potential	13 nm	56
*Allium sativum*	CoCl_2_	Cloves	Fine powdered	Photo-catalytic activity	—	81
Egg white	Co(NO_3_)_2_·6H_2_O		Spongy clumps	Corrosion inhibitor	10–20 nm	166
Starch	CoSO_4_·7H_2_O		Square	Coating application	18 nm	167
Walnut green	Co(NO_3_)_2_·6H_2_O	Walnut green skin	Nearly spherical	—	∼60–80 nm	168
*Cochineal powder*	Co(NO_3_)_2_·6H_2_O	dye	Agglomerate, nanoplate, uniform	Toxicological effect	25–35 nm	169
*Allium tuncelianum*	Co(NO_3_)_2_·6H_2_O	Endemic species	Spherical	Anticancer activity	23 nm	170
Sucrose	Co(NO_3_)_2_·6H_2_O	—	—	Removal of dye	<5 nm	171
Sucrose	CoCl_2_·6H_2_O	—	—	—	30 nm	172
Glycogen					36 nm	
Glucose					23 nm	

## 5. Biomedical applicability of green synthesized cobalt oxide NPs

### 5.1 Antimicrobial activity

Antimicrobial activity refers to the ability to kill or inhibit the growth of microorganisms without harming neighboring tissues. Green synthesized NPs are more effective than those produced by conventional methods due to the presence of secondary metabolites in plants that inhibit microbial growth.^63^ Antimicrobial activities of green synthesized cobalt oxide NPs can vary from plant species to species. There are many methods for evaluating antimicrobial activity by plant extract including disc diffusion method,^127^ well diffusion,^128^ time-kill assay,^173^ agar dilution,^159^ broth dilution methods,^174^*etc.* Out of which disc diffusion and well diffusion methods are the widely utilized methods. There are several types of antimicrobial activity such as antibacterial and antifungal activity which are discussed in this section.

#### 5.1.1 Antibacterial activity

**Fig. 7:**
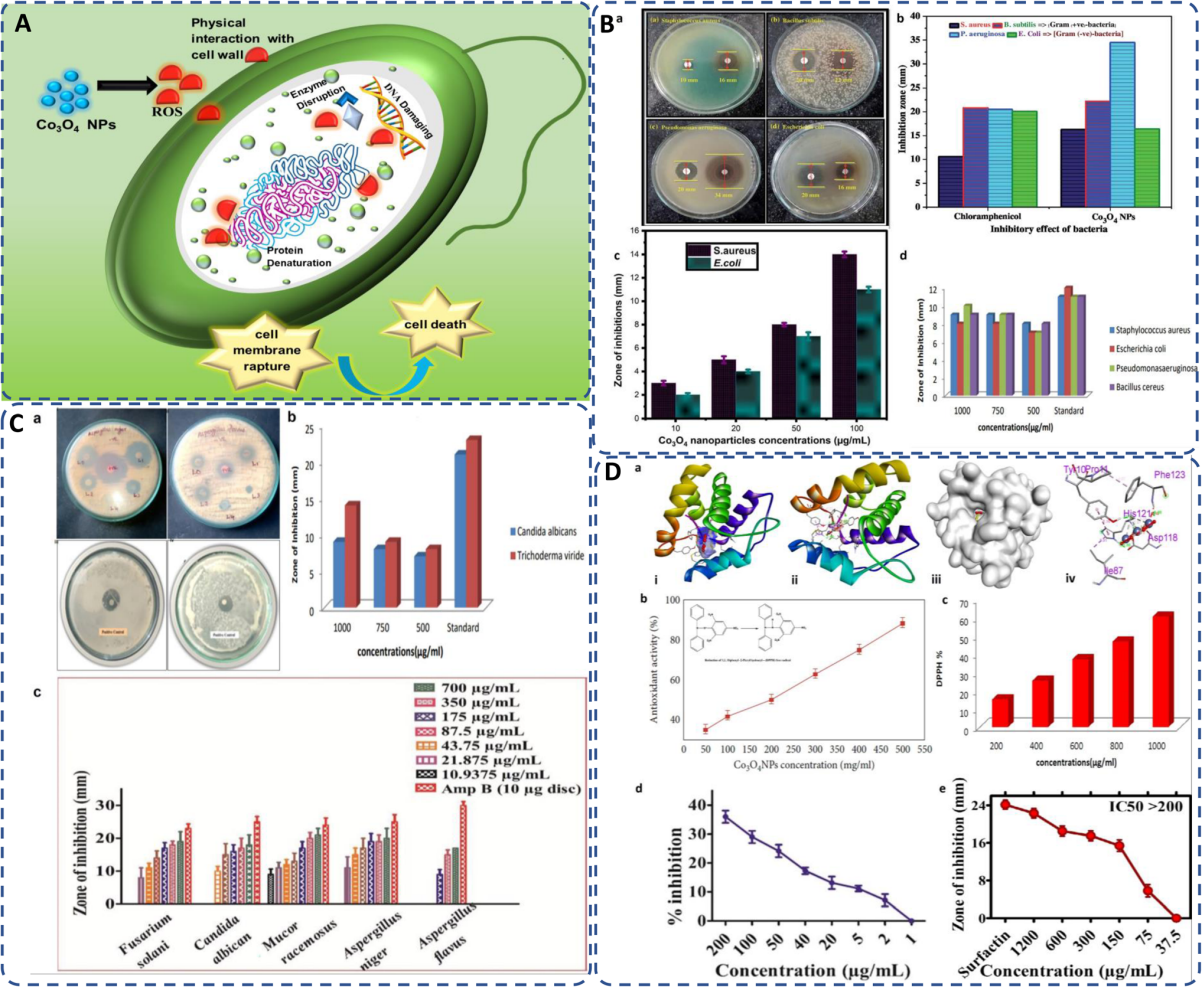
(A) General mechanism of antimicrobial activity of biogenic cobalt oxide NPs causing cell death; (B) antimicrobial activity of green synthesized CO_3_O_4_ NPs against (a) *S. aureus*, *B. subtilis*, *P. aeruginosa*, *E. coli*, (b) inhibitory effect of Co_3_O_4_ NPs in comparison with standard chloramphenicol;^24^ (c) against *S. aureus*, *E. coli* by *Curcuma longa* plant extract;^99^ (d) by *Aerva lanata* at different concentrations.^127^ (C) Antifungal activity of cobalt oxide NPs (a) against *A. favus*, *A. niger*,^139^*A. niger*, *C. albicans*,^147^ (b) against *Candida albicans* and *Trichoderma viride*,^127^ (c) by *Geranium wallichianum* at different concentrations.^26^ (D) (a) Docking studies of green synthesized cobalt oxide NPs showing (i) helical structure, (ii) molecular surface, (iii) 3D display, and (iv) binding interaction with binding sites of 3OGN protein.^21^ Antioxidant activity of biogenically produced cobalt oxide NPs by (b) *Grateloupia sparsa*^130^ (inset reduction of DPPH^82^), and (c) *Aerva lanata* at different concentrations.^127^ (d) α-Amylase enzyme inhibition potential of *Geranium wallichianum* leaves extract mediated cobalt oxide NPs,^26^ and (e) protein kinase inhibition potential of *Rhamnus virgata* leaf extract mediated cobalt oxide NPs.^144^

Bacterial infection is the primary major issue with infectious diseases in terms of death.^175^ Rapid nanotechnology advancements may lead to new compounds with novel antimicrobial properties. Studies on biologically produced cobalt oxide NPs show promising antibacterial results against various strains (Table 7). The Gram-positive bacteria cell wall is ∼70 nm thick peptidoglycan, while Gram-negative bacteria have a 1–2 mm thick lipopolysaccharide layer. Gram-positive bacteria's thinner cell wall ruptures faster, leading to cell death. Cobalt oxide NPs, with a high surface-to-volume ratio, small-sized NPs, interact with bacterial cell walls and generate reactive oxygen species (ROS), which penetrate the cytoplasm and damage the plasmid and nucleus, causing cell death. Fig. 7A illustrates this attack by green synthesized cobalt oxide NPs. For instance, Sabir *et al.* utilized leaf extract of *Phytolacca dodecandra* as a capping or stabilizing agent for preparing cobalt oxide NPs. The study involved disc diffusion method to evaluate antibacterial efficiency of as-prepared cobalt oxide NPs against bacterial strains *E. coli* and *S. aureus*, revealed high zone of inhibition of 10.5, 12.5 mm and 8.3, 11.6 mm at 25 μg ml^−1^ and 50 μg ml^−1^ respectively due to the increased ROS generation as compared to chemically synthesized cobalt oxide NPs.^63^*Aerva lanata* leaves extract utilized for the green synthesis of cobalt oxide NPs showed antimicrobial activities against *E. coli*, *S. typhimurium*, *P. vulgaris*, and *S. aureus* bacterial species by using the disc diffusion method, at different concentrations as shown in Fig. 7B(d).^127^ Green synthesized cobalt oxide NPs *via* plant extract (*Aerva javanica*), bacterial strain (N1C1), and, fungus (*Fusarium oxysporum*) showed their significant antibacterial performance against *S. aureus*, *E. coli*, *P. aeruginosa*, and *B. subtilis*.^85^ Similarly, cobalt oxide NPs synthesized by *Azadirachta indica* leaf extract showed remarkable antibacterial activity against *S. aureus*, *B. subtilis*, *P. aeruginosa* (highest 34.5 mm), and *E. coli* in comparison with standard chloramphenicol^24^ as depicted in Fig. 7B(a). The *Curcuma longa* plant leaves were also utilized for the synthesis of cobalt oxide NPs and displayed their dose-dependent antibacterial activity against *S. aureus* and *E. coli* at different concentrations (Fig. 7B(c)).^99^

**Table 7:** Antibacterial activities of green synthesized cobalt oxide NPs

Source	Test microorganism	Conc.	Method	Zone of inhibition (mm) or MIC (μg ml^−1^)	Ref.
*Hibiscus Rosa sinensis*	*E. coli*	20 mg	Disc diffusion	16 ± 1.13 mm	90
	*P. vulgaris*			21 ± 1.32 mm	
	*P. aeruginosa*			20 ± 1.47 mm	
*Geranium wallichianum*	*P. aeruginosa*	700–21.875 μg ml	Agar disc diffusion	175 μg ml^−1^	26
	*B. subtilis*			21.875 μg ml^−1^	
	*K. pneumonia*			175 μg ml^−1^	
	*E. coli*			43.75 μg ml^−1^	
	*S. aureus*			87.5 μg ml^−1^	
*Mollugo oppositifolia*	*E. coli*	—	Standard agar method	23.6 ± 1.25 μg ml^−1^	21
	*P. aeruginosa*			34 ± 0.21 μg ml^−1^	
	*S. aureus*			28 ± 1.12 μg ml^−1^	
	*B. cereus*			26.56 ± 0.56 μg ml^−1^	
*Sageretia thea*	*P. aeruginosa*	1000 μg ml^−1^ to 31.25 μg ml^−1^	Disc diffusion	250 μg ml^−1^	124
	*S. aureus*			31.25 μg ml^−1^	
	*S. epidermis*			125 μg ml^−1^	
	*E. coli*			31.25 μg ml^−1^	
	*B. subtilis*			125 μg ml^−1^	
	*K. pneumonia*			62.5 μg ml^−1^	
*Phytolacca dodecandra*	*E. coli*	50 μg ml^−1^	Disc diffusion	12.5 mm	63
	*S. aureus*			11.6 mm	
*Grateloupia Sparsa*	*E. coli*	30 μg ml^−1^	Zone of inhibition	11.7 ± 3.2 mm	82
	*P. aeruginosa*			12.5 ± 3.9 mm	
	*B. subtilis*			14.3 ± 3.1 mm	
	*S. aureus*			17.6 ± 4.2 mm	
*Arishta*	*S. aureus*	—	Disc diffusion	30 mm	87
	*S. mutans*			34 mm	
	*K. pneumonia*			40 mm	
	*E. coli*			29 mm	
*Ziziphus oxyphylla* (beri)	*S. aureus*	16 mg ml^−1^	Well diffusion	14.8 ± 1 mm	89
	*E. coli*			23.1 ± 1.2 mm	
*Clitoria ternatea*	*S. thermophilus*	500 μg ml^−1^	—	14 ± 0.7 mm	125
*Calotropis procera*	*E. coli*	750 μg per disc	Disc diffusion	17 mm	111
	*Alcaligenes* sp*.*			13 mm	
	*Enterococcus* sp*.*			14 mm	
	*Pseudomonas* sp*.*			12 mm	
*Curcuma longa*	*E. coli*	100 μg ml^−1^	Well diffusion	∼14 mm	99
	*S. aureus*			∼11 mm	
*Populus ciliata* (safaida)	*B. subtilis*	2, 4, 8 mg ml^−1^	—	21.8 ± 0.7 mm	128
	*B. licheniformis*			18.6 ± 0.8 mm	
	*K. pneumonia*			17.0 ± 0.6 mm	
	*E. coli*			14.0 ± 0.6 mm	
*Sesbania sesban*	*S. aureus*	50 μl (50 μg)	Well diffusion	7.5 mm	130
*Psidium guajava*	*S. aureus*	200 μg ml^−1^	Agar well diffusion	18 mm	131
	*E. coli*			15 mm	
*Citrus limon*	*S. aureus*	—	Agar well diffusion	27 mm	139
	*S. mutans*			28 mm	
	*K. pneumonia*			27 mm	
	*E. coli*			29 mm	
*Rhamnus virgata*	*S. aureus*	1200 μg ml^−1^	Disc diffusion method	∼20 mm	144
	*E. coli*			∼20 mm	
	*K. pneumonia*			∼18 mm	
	*B. subtilis*			∼22 mm	
	*P. aeruginosa*			∼18 mm	
*Vitis vinifera*	*S. aureus*	10 μl of 0.001 g/10 μl	Disc diffusion	—	148
	*B. subtilis*				
	*P. aeruginosa*				
	*E. coli*				
*Mappia foetida*	*S. aureus*	—	Well diffusion	33.5 mm	150
	*B. subtilis*			33.5 mm	
	*E. coli*			38.3 mm	
	*P. vulgaris*			37.3 mm	
*Aspergillus brasiliensis*	*B. subtilis*	5 mg ml^−1^	Agar diffusion	15.6 ± 0.577 mm	159
	*S. aureus*			20 ± 0.288 mm	
	*P. aeruginosa*			11.3 ± 0.577 mm	
	*E. coli*			12 ± 0.288 mm	

#### 5.1.2 Antifungal activity

The cobalt oxide NPs have high antifungal activity, for instance, the NPs formed by the methanol extract of the plant show 47% and 10% inhibition in the growth of *Fusarium moniliform* and *Fusarium solani* fungi, respectively. Similarly, cobalt oxide NPs synthesized by bacteria showed 20% inhibition of *Fusarium solani* growth and fungus-mediated NPs inhibited the growth of *Fusarium moniliform* by 25.3%.^85^ Similarly, the antifungal efficiency of cobalt oxide NPs by using *Citrus limon* fruit was estimated against *A. favus*, and *A. niger* using the agar disc diffusion method and shows 15 and 25 zone of inhibition, respectively^139^Fig. 7C(a). The antifungal performance of green synthesized cobalt oxide NPs was analyzed against *Candida albicans* and *Aspergillus niger* using different fractions of extract of hexane and methanol with plant extract of *W. coagulans* (Fig. 7C(a)).^147^ The *Aerva lanata* plant leaves mediated of cobalt oxide NPs displayed their antifungal activity against *Candida albicans* and *Trichoderma viride* fungal strain at different concentration as shown in Fig. 7C(b), and the zone of inhibition observed at 9 mm and 14 mm at 1000 μg ml^−1^, respectively.^176^ Iqbal *et al.* evaluated the antifungal activity of *G. wallichianum*-mediated cobalt oxide NPs by disc diffusion method against *Aspergillus flavus*, *Candida albicans*, *Fusarium solani*, *Aspergillus niger*, and *Mucor racemosus* at various concentrations as mentioned in Fig. 7C(c).^26^ It is assumed that the cobalt oxide NPs disrupt the protein structure on the surface of the fungal cell, and induce the ROS oxidative stress response. The oxidation of fungal cell lipid membrane is attributed to the free radicals generated by the NPs and the overall inhibition depends on the source and concentration of extract.^140^

### 5.2 Larvicidal activity and docking studies

The parasitology of green synthesized cobalt oxide NPs has been recently carried out by Gowthami *et al.* against urban mosquito larvae *Culex quinquefasciatus*, by using *Mollugo oppositifolia* L. aqueous leaf extract as bioreductant. The biosynthesized Co_3_O_4_ NPs displayed remarkable larvicidal activity, possessing 34.96 μg ml^−1^, LD_50_ value, compared to both the aqueous plant extract and the control, permethrin (Table 8).^21^ Similarly, the docking studies of green synthesized Co_3_O_4_ NPs with larvicidal odorant 3OGN binding protein revealed significant binding affinity of −8.5 kcal mol^−1^ as compared to the control, permethrin (–4.4 kcal mol^−1^). Since, the protein-ligand binding interaction is driven by the hydrogen bonding, therefore the hydrogen donor and acceptor bond distance should be less than 3.5 Å.^21^ The green synthesized Co_3_O_4_ NPs, in this case, exhibit three hydrogen bond interaction with amino acids Asp118, His121, and Phe123 attributing to bond distance, 2.10, 1.63, and 1.98, respectively (Fig. 7D(a)), indicating excellent inhibition ability in larvicidal mosquito 3OGN protein.^21^ Green cobalt oxide NPs exhibit potent larvicidal activity, particularly against mosquito larvae, due to their ability to generate ROS and disrupt cellular function in larvae. Their eco-friendly nature makes them a viable alternative to chemical pesticides, reducing environmental harm while effectively targeting vectors of diseases such as malaria and dengue.

**Table 8:** Larvicidal activity of green synthesized Co_3_O_4_ NPs^20^

Compound	Mortality (%)/concentration (μg ml^−1^)				LD_50_ (μg ml^−1^)
	100	75	50	25	
Aqueous plant extract	60.20 ± 1.64	46.78 ± 0.24	24.16 ± 1.42	10.86 ± 0.84	82.41
Co_3_O_4_ NPs	100 ± 0.00	82.14 ± 1.25	68.48 ± 0.68	32.86 ± 1.20	34.96
Permethrin (control)	70 ± 0.67	46 ± 1.29	30 ± 1.78	16 ± 0.98	72.44

### 5.3 Antioxidant activity

Free radicals, regularly generated in the human body, can deteriorate lipids, DNA, and proteins, causing diseases like cancer and cardiovascular issues.^131^ The antioxidants present in the plant or microbial extract counteract these harmful effects.^177^ Cobalt oxide NPs, as electron donors, stop radical chain reactions by stabilizing free radicals.^82^ It has been observed that DPPH scavenging activity of green synthesized cobalt oxide NPs shows increased antioxidant activity with higher concentrations.^178^ Most commonly used DPPH assay (2,2-diphenylpicrylhydrazyl) tests the scavenging and antioxidant ability of cobalt oxide NPs by eqn (2):2
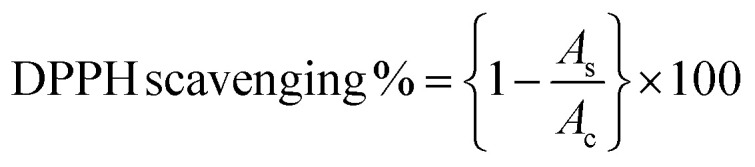
where *A*_c_ and *A*_s_ represents absorbance of control and absorbance of sample respectively. For instance, the antioxidant activity at different concentrations by *Grateloupia sparsa* synthesized cobalt oxide NPs revealed highest DPPH radical scavenging at 500 mg ml^−1^ (88.2%) and lowest at 50.1 mg ml^−1^ (35.0%) (Fig. 7D(b)).^82^ Similarly, *Aerva lanata* leaves extract produced cobalt oxide NPs displayed dose-dependent DPPH radical scavenging activity at different concentrations such as 200, 400, 600, 800 and 1000 μg ml^−1^, of cobalt oxide NPs attributed to DPPH% at 15.07, 25.29, 37.18, 46.73, and 60.46% respectively, as depicted in (Fig. 7D(c)).^127^*Geranium wallichianum* produced cobalt oxide NPs with excellent antioxidant properties observed at 200 μg ml^−1^ with 57.79% reducing power and 89.56% DPPH activity.^26^*Cordia myxa* extracts yielded 46.38% DPPH scavenging at 100 mg ml^−1^.^92^*Sageretia thea* synthesized cobalt oxide NPs showed 57% DPPH scavenging at 200 μg ml^−1^, with total reducing power and antioxidant activity of 19.8 μg AAE per mg and 23.6 μg AAE per mg, respectively, at the same concentration.^124^ Overall, the ability of biosynthesized Co_3_O_4_ NPs to scavenge free radicals is largely attributed to the presence of bioactive compounds from biological extracts used in synthesis. These antioxidants can neutralize oxidative stress, which is beneficial in preventing cellular damage and aging-related diseases.

### 5.4 Enzyme inhibition

Green synthesized cobalt oxide (CoO) NPs have shown potential in inhibiting enzymes that can cause chronic diseases if persistent. The inhibition of the α-amylase enzyme, which helps prevent prolonged high glucose levels that can lead to diabetes, is recently studied. CoO NPs synthesized from *G. wallichianum* showed no inhibition at 1 μg ml^−1^ but achieved 36% inhibition at 200 μg ml^−1^ (Fig. 7D(d)). The study used a reaction mixture containing 25 μl enzyme, 15 μl PBS, 40 μl starch solution, and 10 μl cobalt oxide NPs, incubated for 30 minutes at 50 °C, with distilled water and acarbose as controls.^26^

Similarly, bioinspired CoO NPs from *Rhamnus virgata* leaf extract were tested for inhibiting the cancer-related protein kinase enzyme Fig. 7D(e). While the enzyme itself doesn't cause cancer, it promotes phosphorylation, crucial for metabolic and genetic processes, and its malfunction can lead to carcinoma. The inhibition study using *Streptomyces* 85E fungal strains showed a zone of inhibition up to 18 mm at 1200 μg ml^−1^. The disc method involved incubating 10 μl of CoO NPs on a microbial lawn for 72 hours at 30 °C, with surfactin and DMSO as controls.^144^ Therefore, it suggests that the green-mediated cobalt oxide NPs have potential in managing neurological disorders and diabetes by inhibiting the responsible enzymes.

### 5.5 Anticancer activity

**Fig. 8:**
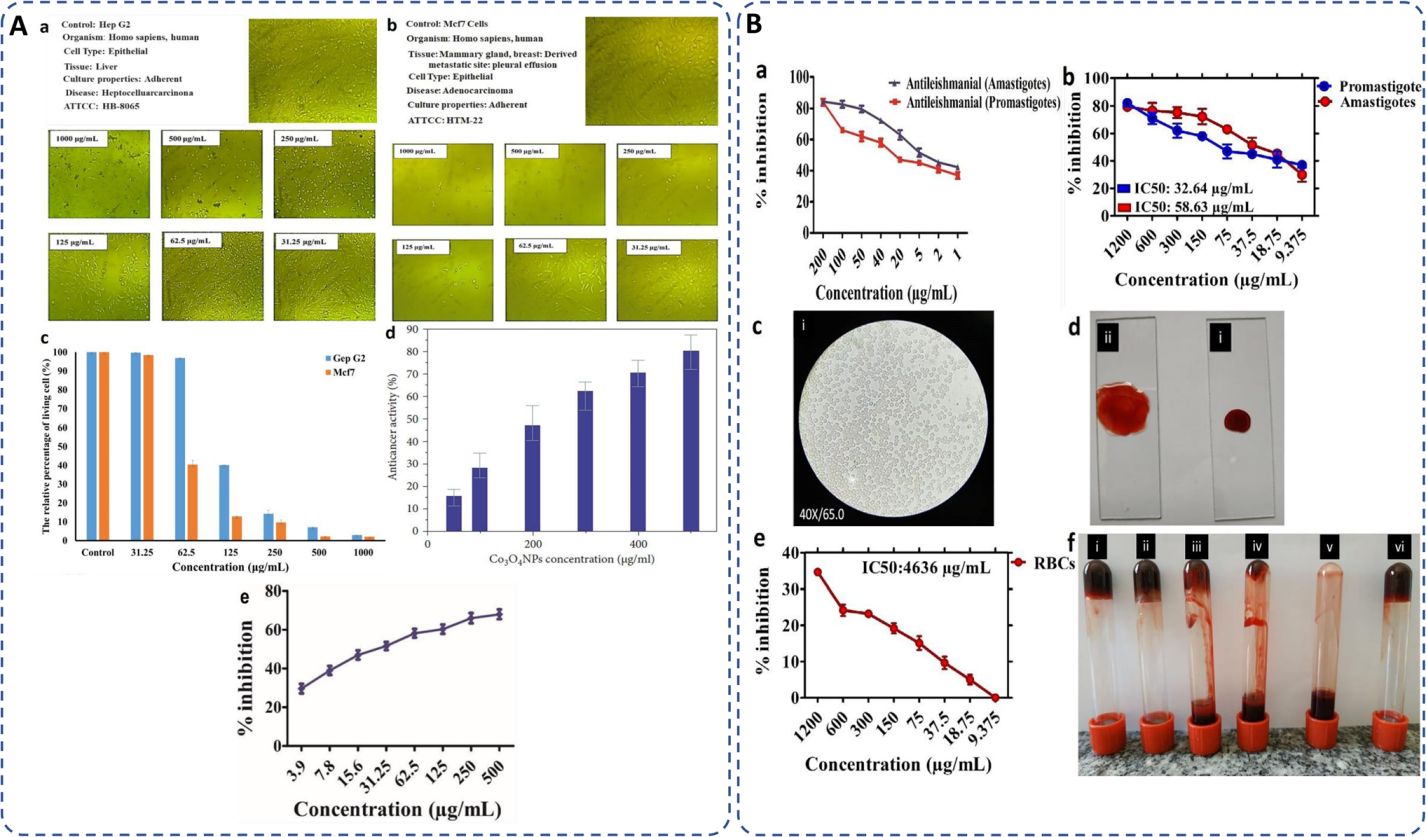
(A) Microscopic images of (a) Hep G2 and (b) Mcf7 cancer cells after treatment with various concentrations of Co_3_O_4_ NPs synthesized using rosemary leaf extract (c) The proportions of viable cells of Hep G2 and Mcf7 cancer cells following exposure to varying concentrations of Co_3_O_4_ NPs produced utilizing rosemary leaf extracts,^20^ (d) anticancer potency of cobalt oxide NPs against HepG2 cancer cell^82^ (e) cytotoxicity against HepG2 cancer cell line.^26^ (B) Antileishmanial activity of (a) *G. wallichianum*^26^ (b) *Rhamnus virgata*-assisted cobalt oxide NPs at different concentrations.^144^ (c) Microscopic images of less hemolytic activity of biosynthesized cobalt oxide NPs in red blood cells.^31^ (d) Thrombolytic activity of biosynthesized cobalt oxide NPs;^31^ (e) haemolytic activity of *Rhamnus virgata* mediated cobalt oxide (CoO) NPs against human RBCs^144^ and (f) anticoagulating behaviour of green synthesized cobalt oxide NPs.^31^

Cancer remains a leading cause of global mortality, with nearly 10 million deaths annually and an estimated economic burden of $25.2 trillion from 2020–2050.^179,180^ Researchers are exploring cost-effective alternatives to traditional anticancer agents, with cobalt oxide NPs emerging as promising candidates due to their lower toxicity,^106^ ferromagnetic properties,^101^ and cytotoxicity.^181^ Studies on Co_3_O_4_ NPs synthesized using rosemary leaf extract showed that at lower concentrations (≤31.25 μg ml^−1^), they were non-toxic to Hep G2 and MCF-7 cancer cells, but at 125 μg ml^−1^, metabolic activity decreased significantly (Fig. 8A(a–c))^20^ while *G. sparsa* mediated NPs showed 80% mortality against HepG2 cells at 500 μg ml^−1^ (Fig. 8A(d)).^82^ Another study on cobalt oxide NPs derived from *Euphorbia tirucalli* plant showed effectiveness against MCF-7 breast cancer cells at different concentrations, with the highest being 100 mg ml^−1^. For this, a 96-well microtiter plate was seeded with 1 × 10^5^ cells per ml at 37 °C and incubated for 24 h with 5% CO_2_ in a humidified environment.^152^ Similarly, *G. wallichianum* leaves mediated cobalt oxide NPs exhibited ∼70% mortality at 500 μg ml^−1^ with an IC50 value of 31.4 μg ml^−1^ (Fig. 8A(e)).^26^ Another study using red algae-derived CoO NPs reported ∼80% mortality at 500 μg ml^−1^ and an IC50 value of 41.4 μg ml^−1^.^31^ Cobalt oxide NPs dissolved in acid present in the stomach having pH around 4.5 and overall, are capable to penetrate cell membranes and dissolve, leading to cell death.^82^ Overall, the green synthesized cobalt oxide NPs demonstrated cytotoxicity against cancer cells. Their small size and surface bioactivity allow them to interact with and penetrate cancer cells more efficiently, inducing apoptosis through oxidative stress and mitochondrial dysfunction. These NPs can potentially be used for targeted cancer therapy with minimal toxicity to healthy cells and additionally warranting further investigation in *in vivo* models, 3D and 4D bioprinting of tissue engineering.

### 5.6 Antileishmanial activity

The capability of a substance to inhibit or kill Leishmania parasites (*Leishmania tropica*), causing the disease leishmaniasis, is referred to as antileishmanial activity. Leishmaniasis affects millions in over 98 countries, with current treatments being costly, toxic, and drug-resistant.^22,182^ Cobalt oxide NPs offer a promising solution due to their antileishmanial properties. Leishmania has a digenetic life cycle, existing as motile promastigotes outside the body and non-motile amastigotes inside the body.^26,124^ Khalil *et al.* for the first time demonstrated a dose-dependent antileishmanial response by *Sageretia thea* plant-mediated CoO NPs along with its vaccination-based prophylaxis.^124^*G. wallichianum* biogenically produced cobalt oxide NPs showed higher activity against amastigotes (IC50: 3.12 μg ml^−1^) compared to promastigotes (IC50: 9.53 μg ml^−1^), with maximum inhibition at 200 μg ml^−1^. The MTT cytotoxic test confirmed higher susceptibility of amastigotes (Fig. 8B(a)).^26^ Similarly, *R. virgata* plant-derived CoO NPs revealed dose-dependent activity, with amastigotes (IC50: 58.63 μg ml^−1^) and promastigotes (IC50: 32.64 μg ml^−1^) when exposed to various concentrations (1200–9.375 μg ml^−1^) for 72 hours (Fig. 8B(b)).^144^ The efficacy of green synthesized cobalt oxide NPs in treating and vaccinating against leishmaniasis is largely dependent on the active chemicals present in the source extract, which determines the IC value. Despite varying IC values, these NPs exhibit a consistent trend of antileishmanial activity. Overall, green synthesized cobalt oxide NPs have shown promise in combating leishmaniasis and hold potential for treating other parasitic diseases which need further exploration.

### 5.7 Haemolytic, anticoagulant and thrombolytic activity

The biocompatibility of cobalt oxide NPs is assessed through hemolytic assays.^31^ Increased hemolytic activity reduces antibacterial efficiency due to erythrocyte membrane interaction with peptide antibiotics, causing hemoglobin leakage.^178^ Materials with hemolysis activity over 5% are considered hemolytic, 2–5% as mildly hemolytic, and below 2% as non-hemolytic, per American Society recommendations.^144,183^*G. wallichianum* leaf-derived cobalt oxide NPs are non-hemolytic at 2 μg ml^−1^, mildly hemolytic at 5–40 μg ml^−1^, and hemolytic above 45 μg ml^−1^.^26^ On comparing the hemolytic potential of cobalt oxide NPs, Trinton-X-100 shows 97.3% toxicity whereas red algae derived NPs shows only 5.3% toxicity.^82^ Similar results were observed by Ajarem *et al.* exhibiting 2.3% toxicity compared to 97.3% for Triton-X-100 and 1.01% for PBS (Fig. 8B(c)).^31^*R. virgata*-derived NPs are non-hemolytic at 9.375 μg ml^−1^ but highly hemolytic at 1200 μg ml^−1^ (IC50: 4636 μg ml^−1^) (Fig. 8B(e)).^144^ So, overall, the green synthesized NPs exhibit dose-dependent least toxicity. Hemolytic activity is calculated as eqn (3):^183^3
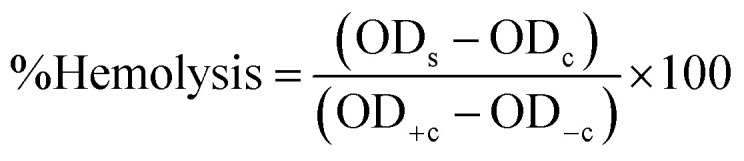
where OD_s_ and OD_c_ are the optical densities of the blood sample and control, and OD_+c_ and OD_−c_ are the positive and negative control, respectively.

Additionally, biosynthesized cobalt oxide NPs possesses anticoagulating and thrombolytic activity. Evaluation studies revealed that blood samples thickened and formed clots in a dose-dependent manner with green-synthesized cobalt oxide NPs. Thrombolytic studies indicated that blood clots dissolved within 30 minutes of adding cobalt oxide NPs (Fig. 8B(d)). Fig. 8B(f) demonstrates that increasing Co_3_O_4_ NP concentration from 10 μl to 50 μl enhanced anticoagulating activity, reaching a maximum at 50 μl.^31^ Overall, green-synthesized cobalt oxide NPs have shown promise in biomedical applications related to blood. Studies indicate that these NPs can exhibit hemolytic activity at certain concentrations, making them a candidate for drug delivery applications. Additionally, their anticoagulant and thrombolytic properties suggest potential for treating blood clot-related disorders, but there is a limited amount of research on the biocompatibility of cobalt oxide NPs. In-depth studies on their anticoagulating and thrombolytic properties, with varying parameters, such as size and stability of NPs, need to be further explored.

Several studies have shown that such metal oxide NPs exhibit different toxicity levels depending on their concentration, route of administration, and the type of cells or organisms they interact with.^79,184^ Instead, green methods help mitigate the toxicity of cobalt oxide NPs. The toxicity of cobalt oxide NPs can indeed be dose-dependent.^79^ In a particular investigation,^111^ cobalt oxide NPs synthesized through a green method were eco-toxically evaluated; the findings indicate that these NPs were predominantly very minimal toxicity and possessed minimal risk to environmental systems. They reported that these NPs exhibit only lower toxicity at significantly high concentrations, specifically at 750 μg per disc, therefore, it is safe for living organisms and can be used for various applications, including in medicines.^111^ Most of the related studies generally do not focus on the amount of toxicity. But on the basis of other studies such as, cytotoxicity, hemolytic studies, we can analyze and develop an idea regarding their toxicity. Table 9 shows a generalized analysis of dose-dependent toxicity of the biosynthesized cobalt oxide NPs.

**Table 9:** Dose-dependent toxicity of green synthesized Co_3_O_4_ NPs

Nanoparticle source	Toxicity dose	Tested organism/cells	Observations	Ref.
*Geranium wallichianum*	2 μg ml	Human erythrocyte and macrophages	Non hemolytic	26
*Geranium wallichianum*	5–40 μg ml	Human erythrocyte and macrophages	Slightly hemolytic	26
*Geranium wallichianum*	>45 μg ml	Human erythrocyte and macrophages	Hemolytic	26
*Rosemary* leaf extract	≤31.25 μg ml	MCF-7 and Hep G2 cancer cells	Non-toxic	20
	≤62.5 μg ml			
*Rosemary* leaf extract	125 μg ml	Hep G2 and MCF-7 cancer cells	Toxic effect	20
*Grateloupia sparsa*	500 μg ml	HepG2 cell line	Anticancer activity (80%)	82
Red algae (aqueous extract)	500 μg ml	HepG2 cell line	Anticancer activity	31
*Nodosilinea nodulosa*	200 μg ml	Brine shrimp	High cytotoxicity	79
*Nodosilinea nodulosa*	50 μg ml	Brine shrimp	Low cytotoxicity	79

### 5.8 Drug sensing

**Fig. 9:**
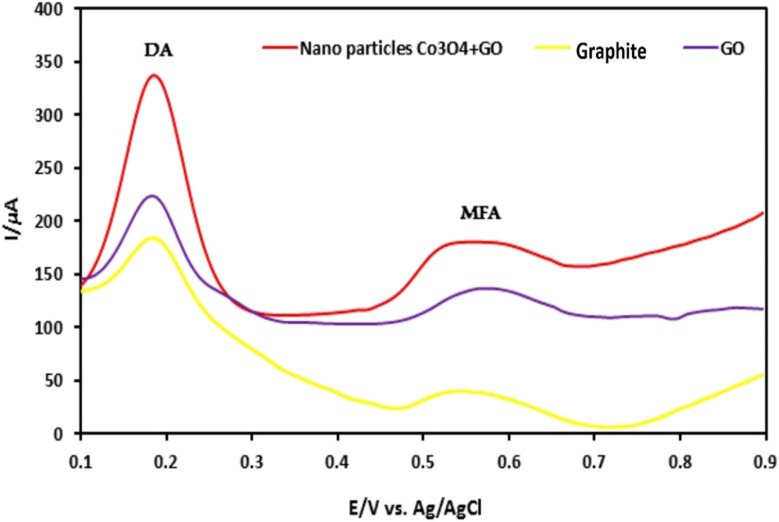
Differential pulse voltammogram (DPV) study showing sensing abilities of *Elaeagnus angustifolia* leaf extract mediated cobalt oxide NPs with GO on CPE for sensing dopamine (DA) and mefenamic acid (MFA).^185^

Sensing activity of sensors is analyzed by calculating the sensors resistance in different environment. Recently, *Elaeagnus angustifolia* leaf extract-mediated cobalt oxide NPs has been explored as a bioreductant and stabilizing agent for drug sensing abilities. Dopamine (DA) and mefenamic acid (MFA) sensing abilities of cobalt oxide (Co_3_O_4_) NPs with graphene oxide (GO) and carbon paste electrode (CPE), were studied with enhancing electron transfer processes for DA and MFA. Under optimized conditions, the electrode showed a linear response to DA (0.5–250 μM) and MFA (1–500 μM) with detection limits of 0.15 μM for DA and 0.3 μM for MFA. Differential pulse voltammetry (DPV) analysis in phosphate buffer (pH 7) identified the best performance with 6% GO and 8% Co_3_O_4_ NPs modifications. The Co_3_O_4_NPs/GO/CPE configuration exhibited the highest oxidation peak currents, indicating superior sensitivity for simultaneous DA and MFA detection (Fig. 9).^185^ Overall, the drug-sensing capabilities of biogenically synthesized cobalt oxide NPs demonstrate their efficacy and potential for broader applications. These NPs can be further explored beyond drug sensing, encompassing areas such as motion detection, conductivity, mechanical properties, pulse monitoring, stress–strain measurement, and pressure sensing on nerves, thus highlighting their extensive applicability in the medical field.

## 6. Enhanced activity of green synthesized cobalt oxide NPs

The green synthesized cobalt oxide NPs influence the controllable morphology, and improve the crystallinity of NPs which can lead to enhanced activity of NPs.^83^ In green methods, biomolecules from plant extracts can bind to the surface of the NPs, introducing functional groups like carboxyl, hydroxyl, or amino groups. These functional groups can enhance the interaction with reactants and promote specific properties.^104^ Plant extracts also contain biomolecules like proteins, enzymes, or polysaccharides, which can act as reducing, capping, and stabilizing agents during NP formation.^131^ These are the reasons behind the enhanced activity of green synthesized cobalt oxide NPs as compared to conventionally synthesized NPs. For instance, Adino *et al.* reported the enhanced antibacterial activity of green synthesized cobalt oxide NPs as compared to other conventionally synthesized cobalt oxide NPs, even at low concentrations (25 μg ml^−1^). This is because, in biologically synthesized Co_3_O_4_ NPs, the generation of reactive oxygen species is significantly high which facilitates the inhibition or kill the bacteria due to the presence of phytochemicals emanating from *Phytolacca dodecandra* (plant extract) and also due to the relatively higher surface area of NPs to volume ratio in case of green synthesized ones.^108^

## 7. Challenges and future perspective

Despite the promising potential of biogenic synthesis methods for cobalt oxide NPs, several challenges need to be addressed for widespread application and scalability. One significant challenge is optimizing synthesis protocols to ensure reproducibility, uniformity, and control over the size, shape, and properties of the synthesized NPs. Variability in the composition of natural extracts and microorganisms can lead to inconsistencies in NPs synthesis, necessitating standardized protocols and rigorous quality control measures. Elucidating the underlying mechanisms governing biogenic NPs synthesis is another challenge. The precise interactions of bioreductants, reducing agents, and phytochemicals in NP formation remain poorly understood. Further research is needed to unravel these complex mechanisms and optimize synthesis conditions to improve yield and properties of cobalt oxide NPs. Scalability presents another challenge, especially for large-scale biomedical applications. Innovative research approaches, interdisciplinary collaboration, and concerted efforts from academia, industry, and regulatory bodies are essential to overcome these challenges.

Although cobalt oxide NPs have shown promise in various biomedical applications, optimizing their biocompatibility is essential. Future research should focus on minimizing cytotoxicity through controlled synthesis techniques, ensuring safer interactions with biological tissues. The intrinsic antimicrobial and anticancer activities of cobalt oxide NPs are promising, future research coupled with bio-inspired synthesis methods with predictable mechanism, could enhance their therapeutic efficacy and promote their use in clinical applications.

Beyond antimicrobial and anticancer studies, more research is required to explore biological interactions such as drug delivery, biosensing, and protein binding. For targeted drug delivery, cobalt oxide NPs can be engineered for targeted drug delivery systems by functionalizing them with specific biomolecules. Continued advancements in surface modifications and smart nanocarrier designs could improve their efficiency in delivering drugs to diseased cells while reducing side effects. The development of multifunctional cobalt oxide NPs for theranostic applications, combining therapeutic and diagnostic capabilities, holds considerable promise. Future research should explore the integration of imaging, drug delivery, and therapeutic properties into a single nanoplatform for real-time disease monitoring and treatment.

Additionally, the efficacy, safety, and long-term stability of biogenic cobalt oxide NPs need comprehensive toxicity studies and *in vivo* assessments. Concerns related to biocompatibility, cytotoxicity, and potential environmental impact must be addressed to ensure the safe and responsible use of these NPs in tissue engineering, 3D bioprinting, biosensors, organ on chip and real-world applications. By addressing these issues, we can unlock the full potential of biogenic cobalt oxide NPs, harnessing their unique properties for critical societal, environmental, and technological applications.

## 8. Conclusion

Overall, the synthesis of cobalt oxide NPs *via* green and sustainable methods represents a significant advancement in nanomaterial synthesis particularly in biomedical applications. By exploring natural resources and harnessing the unique capabilities of plant, its parts, algae, fungi, and microorganisms as reducing and stabilizing agents, researchers can synthesize cobalt oxide NPs in a sustainable, eco-friendly, and cost-effective manner by circumventing the limitations associated with traditional synthesis methods. The biological source plays a vital role in determining the reducing efficacy, size and stability of the NPs resulting into their effect on biocompatibility. Since there is a gap and large avenue for diverse studies and factors affecting their biocompatibility which need to be explored. Therefore, we have highlighted the diverse sources, methods, probable mechanism involved in the biogenic synthesis of cobalt oxide NPs, emphasizing their eco-friendly attributes, by sophisticatedly analysing their structure, morphology, size and stability, and their biocompatibility potential for biomedical applications. The biogenically derived cobalt oxide NPs shows protean performance in biomedical field, including antibacterial, antifungal, larvicidal, antioxidant, enzyme inhibition, anticancer, antileishmanial, hemolytic, anticoagulant, thrombolytic activities and drug sensing ability. Therefore, the convergence of green synthesis approaches with nanotechnology holds great promise for advancing sustainable practices, addressing environmental, and adaptative challenges, and paving the way for the development of innovative environment benign solutions for the future medical approaches.

## Data availability

No primary research results, software or code have been included and no new data were generated or analysed as part of this review.

## Author contributions

Annu.: conceptualization, investigation, formal analysis, writing – original draft preparation, writing – reviewing and editing, visualization. Muskan Sahu: investigation, writing – original draft preparation. Somesh Singh: investigation, writing – original draft preparation. Satypal Prajapati: investigation, writing – reviewing and editing. Dinesh K. Verma: supervision. Dong Kil Shin: supervision, resources.

## Conflicts of interest

The authors declare that there is no conflict and competing interest that could have appeared to influence the paper.
